# Part III. Clinical Review and Hospital Reports

**Published:** 1833-07-01

**Authors:** 


### Clinical Review and Hospital Reports.

I. Report of Cases treated in the Surgical Wards. By John Stirling, Surgeon to the Royal Infirmary, &c. Two Quarterly Reports.*

[Glasgow Royal Infirmary.]

Reports of surgical practice are furnished with more regularity by the surgeons of the Glasgow Royal Infirmary, than by those of any other institution with which we are acquainted. We have, on many occasions, had the pleasure of noticing them in terms of commendation, and if any remarks of ours have contributed to their steady publication, we need scarcely say that we must experience a high degree of satisfaction. We have dwelt so often and so long on the benefits that must accrue to science by a steady and well regulated system of clinical reports, that we will not take up our readers' time by reiterating our arguments, our expostulations, or our advice. If hospital surgeons and physicians will not engage in an extensive system of hospital reporting it is no fault of ours, and we venture to observe, that they ill consult their own best interests by holding back.

As an encouragement to those who do communicate to the public the results of their hospital experience, and as a record of the valuable facts that are related, scanty and imperfect as they are at present, we have resolved to dedicate a considerable portion of our Periscope to hospital reports. We trust that we shall find ere long, a growing inclination to meet the popular demand, and that metropolitan physicians and surgeons will follow the example that has been set them in the provinces, and the distant departments of the empire. To our provincial brethren we have little to say. The zeal, perseverance, and talent now displayed by them, render exhortation almost unnecessary. We trust that they will continue to exert themselves; we trust that by their book societies and associations they will cultivate still further a taste for medical literature, and that by publishing Transactions and Hospital Reports, they will in turn enrich that literature and improve their science. In proportion as they become more generally intelligent, they will demand more unanimously and effectually better institutions, and with better institutions still further improvements will come. Great as has been the progress of the profession within the last half century, we do not doubt that it will continue to advance, and that medical men will occupy a higher and a prouder station in society. But all substantial benefit must be founded on increased knowledge, and this can only result from its free and wide circulation. To this point therefore we return, that those who have opportunities of studying nature on an extensive scale are bound by a regard for science, and by a true acquaintance with their own interests, to communicate their experience to their less fortunate brethren. The periodical press must be their engine. We pass to the consideration of the cases before us.

Case 1.—*Foreign Body in the Trachea—Tracheotomy—Death.*

E. B. æt. 7, admitted March 15th, 1832.

Two hours previous to his admission a small pebble, which he had in his mouth, slipped into his throat; immediately afterwards he was seized with a paroxysm of dyspnœa, amounting almost to suffocation. On admission the respiration was quick but tolerably free—short abrupt cough—on slightly compressing the trachea immediately below the cricoid cartilage, and desiring him to cough, a sharp impulse communicated to the finger, as if some foreign body were impelled against it. Previously to admission a probang had been introduced into the stomach. Ow-

* Glasgow Journal, Nos. I. and II. Vol. I. New Series.

ing to a difference of opinion in consultation, nothing was done till the next day, when the operation of tracheotomy was performed.

An incision was made through integuments, commencing at lower margin of thyroid cartilage, and carried downwards to the extent of an inch. The muscles of the windpipe were large, and the struggles of the boy rendered the operation somewhat difficult. The cricoid cartilage and two rings of the trachea were divided, a small portion of the latter being cut out. By this means a very free opening was made into windpipe. A small artery was divided, and bled freely; a quantity of the blood having entered the trachea, produced some degree of convulsive coughing, which, however, was not followed by the discharge of the foreign body. On introducing a curved probe into the opening, and desiring the boy to cough, the pebble could be felt distinctly impelled against the instrument; but it never ascended so high as the artificial opening. Several unsuccessful attemps were made to get hold of it; it was therefore deemed expedient to remove the boy to bed, and to trust to time for the expulsion of the stone.

In the evening, he had fever and severe pain in the right side of the chest, increased on inspiration or coughing. Six ounces of blood were taken from the arm, with immediate relief. In the afternoon of the 17th, a violent fit of coughing came on, and a small white pebble, of a rounded form and polished surface, was expelled. The edges of the wound were now brought together with adhesive plaster. The throat was slightly painful; there was cough, mucous râle in the upper part of the chest, and pyrexia. *Cal.* gr. ij. 4*tis horis. V.S. ad* ℥vj. *if cough or pain urgent.*

"18th. About six o'clock last night, was seized with a violent paroxysm of dyspnœa. Dressings removed from artificial opening, and double-curved wire introduced, after which he breathed easily and expectorated a quantity of mucus. Complained at that time of pain in situation of glottis, aggravated by lateral pressure. No stool from calomel; a dose of senna was given, and six leeches applied to lower part of neck. Slept well, and had no return of dyspnœa until this morning, when breathing became again difficult, but was immediately relieved by the removal of a quantity of mucus which had collected around the artificial opening. At present breathes easy, and has no pain in chest or upper part of throat. Edges of opening slightly swollen and coated over with lymph; skin warm and moist. Bowels freely opened. Pulse immediately before visit 120, at present 140.—*Calomel to be continued, and leeches repeated if necessary.*"

On the 19th he was relieved, but had some tenderness in the region of the glottis. There was a croupy sound on coughing. The edges of the wound were again brought together. A cough mixture prescribed. He continued to improve till the 21st, when he had a fit of dyspnœa, with livid countenance. The artificial aperture was re-opened and mucus expelled, with great relief. In the evening he was still better. The mouth was affected by the calomel. On the morning of the 23d, after a severe fit of coughing, he became insensible, with contracted pupils, &c. Notwithstanding the usual means were adopted, he died in the forenoon of the 24th.

CASE 2.—*Tumour in the Neck—Laryngotomy—Death.*

David Clarke, æt. 19, admitted June 12th, 1832.

Occupying nearly whole of right lateral region of neck is a large pretty firm tumour, somewhat of a triangular shape, the base extending from middle of body of lower jaw to a point two inches behind ear, and the sides converging from each end of this line to the apex, situated at posterior border of sterno-mastoid muscle, about two inches above clavicle. Tumour is not very moveable, and it seems to dip deeply behind angle of jaw, and to be in some degree attached to it. On opening the mouth, the soft palate is seen thrust forward apparently by some part of the tumour. It is very tender to pressure, but most painful on inside of right ear, the pain shooting up to vertex of head. Deglutition is in some degree impeded, but respiration little affected. Two months ago tumour commenced as a small hard swelling at right side of thyroid cartilage, which has gradually increased in size since that time. General health good, but pulse very irregular.

Blisters were applied without benefit. The tumour continued to increase slowly, and, at 10, p. m. of the 24th, he was seized with suffocative dyspnœa. An incision was made into the tumour, below the lobe of the ear, where it felt boggy. No relief was obtained, and another incision was made below the angle of the jaw; a little blood only escaped. The larynx was then opened between the thyroid and cricoid cartilage, and the edges of the opening kept asunder by a double-bent wire. Some blood getting into the trachea occasioned suffocative cough, but the hæmorrhage ceased spontaneously.

Iodine was given without benefit; the dyspnœa was sometimes better, sometimes worse. An incision was made over the base of the jaw, and a few drops of ill-conditioned pus escaped; the artificial wound was kept open. On the 5th, there was a paroxysm threatening suffocation. A canula, two inches in length, was several times introduced through the wound, but always produced suffocative cough. A tube three-fourths of an inch was tried, but, from its shortness, this was constantly thrown out. A long tube was, therefore, again tried, and, after violent struggling, the patient was able to retain it, and breathed easily. On the 17th he had again an attack of dyspnœa, and on the 22d he died.

On inspection, the heart was found much enlarged and the lungs full of tubercles, some of them suppurating. The tumour appeared to have been originally an enlargement of the right side of the thyroid gland, and was different in structure from any Mr. Stirling has met with. The opening in the larynx was free.

Case 3.—*Wound of Throat—Pharynx opened—Recovery.*

Robert M'Farlane, æt. 54, admitted Dec. 25th, 1832, 7, p.m.

On the previous night he attempted suicide by cutting his throat with a razor. A considerable quantity of blood had been lost, and a vessel had been tied. On admission, the wound is found to be about three inches in length, and its edges are somewhat ragged and irregular. Centre of incision is at the space between the os hyoides and thyroid cartilage, and from thence it extends backwards and obliquely upwards on each side, to within a small distance of the margin of sterno-mastoid muscle. At its centre the incision penetrates very deeply, and lays freely open the pharynx, but becomes more superficial towards each extremity. Is able to articulate pretty well, and can also swallow fluids, although with some difficulty. Has a frequent troublesome cough, with which he was afflicted some time previous to infliction of injury. General health seems good. Pulse 84, of tolerable strength. Tongue white. Bowels slow. *Two stitches, with adhesive plaster and bandage. Cough mixture—anodyne.*

On the 26th, every attempt to swallow liquids produced convulsive cough, and only a very small quantity could be got down. *A pint of beef-tea introduced through the œsophagus tube every six hours.* On the 27th the dressings were removed. No adhesion had taken place. On any attempt to swallow, a severe paroxysm of coughing was induced, and the fluid was ejected at the wound in the throat. The tube could be introduced with facility, and with very little irritation, into the stomach. The symptoms were in all respects favourable. The edges of the wound were again approximated—attempts at swallowing prohibited—and beef-tea introduced through the tube, as before. On the 30th, there was some inflammation of the skin of the anterior part of the neck and superior part of the thorax. *Twelve leeches—two ounces of mag. sulph. injected into stomach.* Next day there was still much tumefaction, and, on removing dressings, a very large coagulum was withdrawn from the wound. Nothing untoward occurred after this, and on February 8th, the patient was dismissed cured.

CASE 4. *Carcinoma of Rectum—Operation—Recovery.*

Alex. Campbell, æt. 28, admitted Nov. 14, 1832.

For nearly an inch round the verge of the anus was a circle of unhealthy looking ulceration, with high everted edges, and a rough callous surface, discharging copiously a thin unhealthy matter. On going to stool there was great pain and occasionally slight bleeding; when the fæces were liquid they passed out involuntarily. The general health was somewhat impaired. The disease had begun a year previously with what appeared to have been hæmorrhoidal tumors. These were excised by a surgeon, the wound did not heal, and the present sore was the consequence. A suppository and chlorine wash was tried without benefit, and the following operation was resorted to.

"The patient being placed in the ordinary posture for lithotomy, and the urine having been previously discharged, a sound was introduced into the bladder to elevate the prostate gland. An incision embracing diseased parts, and extending through skin and considerable part of cellular substance, was then made. Anteriorly the gut was separated from its surrounding connexions by cautious dissection; laterally and posteriorly the knife was passed up the depth of the disease, and its point made to penetrate the walls of the rectum. It was then carried round a considerable portion of the circumference of the gut, guided by the finger introduced into the rectum. The greater portion of the disease being thus removed, it was found that several parts of diseased intestine still remained behind. These were subsequently dissected out along with several glands, which lay internal to the skin, and were enlarged and indurated. Their cellular connexions being pretty strong, they were removed by the finger with some difficulty. Still more difficulty was found in removing some which were firmly adherent to the right lateral lobe of prostate gland. The diseased parts were in this manner removed as far as could be ascertained. The interior of the rectum felt smooth, and its walls soft and pliable. A considerable quantity of blood was lost during the operation, and this escaped from an infinity of small vessels, the greater number of which speedily retracted, a single vessel only requiring a ligature. At one time during the operation there was an approach to syncope, but the pulse never disappeared at the wrist. The cavity formed by excision of diseased parts was filled by several pieces of sponge, and a compress applied externally, supported by a T bandage."

In the night there was vomiting, relieved by effervescing draughts and the application of turpentine to the epigastrium. On the 10th the skin was hot, the pulse 144, but there were no other unpleasant symptoms. The sponge had distended the wound, to which it was adherent. Ordered, *castor oil—antimonial wine—oiled lint to wound.* On the 12th he was ordered quina and tincture of gentian; on the 14th a beef-steak and porter. On the 22d cicatrization was beginning to take place. During January he continued to improve, and the wound to contract, in consequence of which it was necessary to introduce a bougie frequently, to keep the rectum open. Early in February he had an attack of abdominal irritation, with rigor and pyrexia. It was subdued by leeches, laxatives, and effervescing draughts. At the date of report, January, 1833, the wound was nearly healed, but the bougie was still necessary to prevent undue contraction. The fæces could be retained except when very fluid, in which case a small quantity sometimes escaped involuntarily. His general health was tolerably good.

We are not convinced of the propriety of denominating this disease carcinoma. The patient was young, the history was not such as is usually obtained in cases of cancer of the rectum, nor was the situation the ordinary one for that malady. Mr. Stirling gives no account of the structure of the part removed, and we repeat that, taking all these circumstances into consideration, we do not feel assured that the term carcinoma is correct. But if it be so, we need hardly observe, that the patient's permanent recovery is not certain. We must wait before we can pronounce on that point, and we trust that, as the operation of removing the lower portion of the rectum is now exciting interest among surgeons, Mr. Stirling will take some future opportunity of acquainting the readers of the Glasgow Journal with the result.

Mr. Stirling relates a case of what is denominated elephantiasis of the labium pudendi, of a female aged 24. The tumor had originated two years previously from a blow upon the part. It was removed by two semi-elliptical incisions, and there was a good deal of bleeding. The tumor weighed four pounds and seven ounces; it presented internally a gelatinous appearance, and contained a great quantity of serum. The operation was performed on the 15th July, and the patient was dismissed cured on the 9th August.

### Injuries of the Head.

Case 1.—*Scalp-wound, "Arachnitis"—Recovery.*

R. A. æt. 9, admitted May 5, 1832, having received a violent blow on the back of the head with a bunch of keys, ten days previously.

There was a wound on the occiput half an inch in length; bare bone was felt with the probe. There was headache, which had existed since the blow, most severe in the forehead—sluggishness of pupils—occasional fits of sickness, followed by flushings and partial perspirations—heat of surface—pulse 130—tongue foul—bowels constipated. The treatment previously employed had been straps to the wound and purgatives. The treatment employed was shaving the scalp—leeches—a blister—and purgatives. On the 9th, the patient, not being much better, was ordered two grains of calomel and half a grain of opium night and morning. On the 21st the mouth was slightly affected with mercury. The headache had ceased—the scalp-wound was nearly well. There was, however, considerable œdema of the face, and a tendency to the same thing in the scrotum; pulse 120. He was bled to 6 ozs. and purged, and on the 31st the œdema was gone. He was then ordered quina, and on the 5th June dismissed cured.

We mention this case as illustrative of the good effects of mercury in the symptoms of membranous inflammation that follow injuries of the head. We do not think that it is employed so often or so early as it should be, for it not only exerts a power over those symptoms that mere depletion and purgation do not, but it also prevents the necessity of extreme depletion. We should rather be inclined to avoid the combination of opium with calomel in these cases.

Case 2. *Fracture of the Skull—Trephining—Dura Mater punctured—death.*

P. M. æt. 32, was picked up by the police in the streets, on the evening of the 9th July, 1832, and admitted on the 10th, at noon. No history could be obtained.

Insensibility. Respiration very laborious. Pupils fixed, that of right eye very much contracted, left considerably dilated. Pulse about 70, labouring. Heat of surface natural. Immediately over left lateral region of frontal bone, near to its junction with parietal, is a small triangular wound. Bone not denuded, and no fracture or depression apparent. Nearly in same situation on right side, there is considerable tumefaction, but no abrasion of surface. Face is much swollen, particularly about eyelids, which are of a dark livid colour. No particulars of the accident can be obtained, having been picked up by the police on the previous evening. He was immediately bled to 20 ozs. and had a common enema.

By advice of consultation, wound was enlarged by means of a crucial incision, and the angles being reflected back, a fracture was detected in parietal bone running along the superior boundary of the temporal fossa, and extending downwards upon that portion of the frontal bone which enters into the formation of that fossa. Little or no depression of the bone existed, but a little blood oozed from the fracture. Trephine was applied so as to embrace a segment of both sides of fracture, by which a circular piece of bone was removed. The dura mater was found detached to a considerable extent from the skull, more particularly in the direction of the orbit, and when separated from the bone by the intervention of any instrument, a considerable quantity of fluid blood escaped. To facilitate its exit, a triangular piece of bone was removed by means of Hey's saw; the base of the triangle corresponded to the opening made by the removal of the circular portion, and one of its sides to the fracture; the apex projected downwards into the temporal fossa. On removal of this portion, more blood escaped. On pressing upon the dura mater, fluctuation was distinctly felt. An opening of about half an inch in length was made, and a quantity of serum spouted out, at first clear and nearly colourless, but afterwards mixed with red particles of blood. No coagula escaped. Patient was evidently much relieved. Respiration, from being stertorous, became easy and natural. The edges of wound were loosely approximated, he was removed to bed, and cold water directed to be applied to head, and a turpentine enema administered.

At 7, p. m., he was worse—skin hot —pulse 168; considerable discharge from the wound, flowing evidently from anterior part of cranium. He was bled to ten ounces. He died at 10, p. m.

On examination, sanguineous effusion on the upper and lateral surface of right hemisphere, with slight effusion below arachnoid. Great effusion at basis—veins distended—quantity of serum in lateral ventricles. The fracture began at the anterior inferior part of left parietal bone, traversed the orbital process of the frontal and the æthmoid, and involved slightly the right orbital process of the frontal, as well as the body and wings of the sphenoid. The dura mater was not lacerated.

This case shews how futile is the precept, that in concussion the pupil is affected one way and in compression another. In this case one pupil was contracted, the other dilated, although there was such extensive compression.

Case 3. *Compound Fracture of the Skull—Operation—Death.*

A. S., æt. 30, admitted Jan. 10th, 1833, at 1, p. m., having been thrown among stones, in the morning, whilst riding at full gallop. He was said to have vomited some blood. Insensible —breathing somewhat stertorous—pupil of right eye dilated, of left contracted, of both insensible to light—pulse 64, feeble—extremities cold. Above right eye-brow a transverse wound, about three inches in length, extending down to the bone, and a fracture with depression.

Wound of forehead was enlarged, and another incision made at right angles with the former, and carried upwards for the space of an inch. The two angular flaps thus formed, were dissected upwards, and a portion of bone exposed. Crown of trephine was applied over upper margin of fracture, so as to embrace depressed bone. Circular portion being removed, two or three irregular spiculæ were extracted. Taken collectively, these formed a triangle, the sides of which did not exceed three-fourths of an inch. Another portion, about half an inch in length and three lines in breadth was slightly depressed. This portion was elevated to the level of surrounding bone, and allowed to remain. Dura mater was found entire, but separated from the bone to a considerable extent, and a probe could be easily introduced as far as orbitar process of frontal bone. A small quantity of fluid blood issued from the opening. Flaps were brought together and held in contact by plaster and light dressings.

At 7, p.m., he seemed more sensible, and moved the left arm freely—dilatation of right pupil less perceptible—both contracting sluggishly on the approach of light. He had repeatedly vomited grumous blood since the operation. No stool—pulse 66.—*Turpentine enema.*

Next morning the sensibility seemed yet farther increased—both pupils more natural—no attempt to move the right arm, but both lower extremities equally drawn up. No more vomiting—no discharge of urine—pulse 60. Between two and three pints of urine were drawn off by the catheter. In the afternoon the lips of the wound, being adherent, were separated by the probe, and a small quantity of blood escaped. *Cold lotion and turpentine enema.* On the 12th there was rather less sensibility—great difficulty of swallowing and occasional hiccup—urine only voided by catheter—pulse 68, stronger. *Hirud.* xx. *temp. Vesic. capiti—Enema.* On the 13th the pulse was 84, softer. *Hirud.* xij. *temp. Cal.* gr. ij. 2*ndis horis.* In the evening the pulse becoming stronger, he was bled to two pints, when it rose from 84 to 132, and became soft. Next morning he was rather more sensible—left angle of mouth rather retracted—catheter still necessary—pulse 124—edges of wound apart, and a small quantity of pus discharged from it. He was again leeched, after which he grew more sensible and spoke. On the 15th he was less sensible and more feeble—skin cold—pulse 120.—*Half an ounce of brandy every two hours, till the pulse becomes firmer—beef-tea.* On the 16th the mouth was affected and he had purging. On the 17th he died.

On removing the scalp, the space in the skull from which circle of bone had been removed, was found filled with purulent matter. On removing scull-cap, the surface of the dura mater was found natural in appearance, with the exception of that portion above which the trephine had been applied, which was covered with a layer of coagulable lymph, seemingly organized. On raising dura mater a very extensive extravasation of blood was found to have taken place upon the surface of the brain, particularly on the posterior and opposite surface from that which was nearest the fracture. The extravasation was also very copious at the base of the brain. The fracture extended down the frontal bone, and across the orbitar process, in the direction of, and ending seemingly at the base of the crista galli.

Our readers are aware that the surgical treatment of compound fracture of the cranium has of late years attracted considerable attention. Sir Astley Cooper and Mr. Brodie are advocates of immediate trephining in this accident, whilst others oppose the practice, and consider that the immediate application of the trephine is not more necessary in this than in other injuries of the head, or in cases of simple fracture. It is obvious that such a question can only be decided by facts, and therefore we have noticed all the cases of compound fracture of the skull that have met our eye in the periodical or in other publications.

It is obvious that in the preceding case the operation could not prevent, nor was it likely to cause the fatal termination. The operation is resorted to on the principle that portions of bone pressing on or penetrating the dura mater, are more calculated to irritate and produce inflammation of that membrane, than if the depressed portions be raised or removed. But this patient died from the extent of the extravasation and consequent injury to the brain.

### Excision of the Elbow-Joint.

Two cases of excision of this articulation are to be found in the reports before us. We have remarked on a previous occasion, (we allude to our review of Mr. Syme's work, upon Excision,) that it cannot fail to strike the practical reader, how frequently excision of the elbow-joint has been performed, and how rarely amputation for disease of that joint is witnessed. If excision be a substitute for amputation, it follows of course that either excision is performed too often, or amputation not often enough. We must confess that we should feel extremely loth to amputate for disease of the articulation of the elbow, the patient getting off, in the majority of cases, with a stiff-joint. The advocates of excision may reply, that if their operation was resorted to the patient would have an useful instead of a stiff-joint. To this we can only say, that it is not certain which is the better of the two, an elbow-joint anchylosed at a convenient angle, and a strong limb, or a sort of articulation and a weak limb. Then there are undoubtedly some risks run in the operation of excision, although they are neither numerous nor formidable. The question can only be decided by facts. We will give as succinct an account as possible of those before us.

*Case* 1. F. D. æt. 11, of strumous constitution, admitted June 24th, 1832.

Left elbow-joint much swollen—sinuous openings, leading to roughened bone—integuments around them livid—little pain—motions limited. Firm swelling on the dorsum of the foot, chiefly over the third metatarsal bone—a sinuous opening, tending towards this bone, which is not felt bare. Appetite defective—slight cough—occasional night-sweats—pulse 120—bowels irregular. The disease of the elbow joint had begun three months previously—disease of the foot subsequently to the former. He was ordered quina, with acid, and a beef-steak for dinner.

On the 19th, the operation of excision was performed. We need not describe the steps of the operation, so many descriptions of it having lately been given. The lower end of the humerus and the upper ends of the radius and ulna having been removed, all the diseased bone appeared to have been taken away, with the exception of a very small part of the posterior aspect of the ulna. Nothing untoward occurred during the progress of the operation, or afterwards. About the middle of January he had a febrile attack, followed by pain, swelling, and discharge on the dorsum of the foot. On examining the wound there, the middle and external cuneiform bones were found rough, and the probe could be passed into their articulation with the navicular bone. Only one point of the arm remained unhealed, and that was superficial, but several small sores had appeared on the site of the former cicatrices. Under salines, with a recurrence to wine, &c. on the subsidence of pyrexia, the patient again improved, and was "dismissed cured" on the 4th March.

We should have liked a little more specification in the account of the termination of this case. We are told nothing of the nature of the false articulation or the anchylosis—nothing of the powers of the limb—nothing of the state of the foot. And we would draw attention to the latter point. There was disease of the cuneiform bones, and of their articulation with the navicular, and yet, under proper general treatment, the boy is described as being cured. Why not have tried a similar plan in the instance of the elbow-joint. In our English hospitals we Southrons do this—we apply splints and give tonics, and send the patients to the seaside; nor are the results of our treatment discouraging. But in Scotland excision is performed.

*Case* 2. J. A. æt. 29, admitted Sept. 20th, 1832. There is considerable soft elastic swelling round left elbow-joint, extending slightly up arm, and down over upper third of fore-arm. Has a good deal of pain on pressure over condyles of humerus, but more over head of radius. Motions of joint are limited and painful, it admits of very little flexion, and he cannot supinate hand. Does not complain of much pain on approximation of articular surfaces. At inner aspect of fore-arm, a little below joint, swelling is slightly pointed, and seems to communicate a feeling of fluctuation. A sinus which existed at inner side of arm has been closed for some days. General health pretty good—pulse rapid—night-sweats formerly, but not now. The disease is of two years' standing, and originated after exposure to cold. The pain and swelling were aggravated by an injury, and the manipulations of a bone-setter four months ago. On the 23d, a probe, introduced into the opening above the elbow-joint, detected bare bone. He was ordered a blister, quina, and beef-steaks. Suppuration occurred, and increased about the joint, and two free openings were made. The general health became impaired, and, on the 14th Oct. excision was performed. The disease "appeared to be confined to the surface of the bones invested with cartilage." On the next day he had cough—some fever—tongue dry and red. We need not give the particulars of the after-symptoms or after-treatment, suffice it to say, the thoracic affection increased, and the patient died on the 22d of November.

On examination, the pleuræ were found united by old adhesions—the lungs studded with tubercles in their various stages, and presenting cavities in the superior lobes. The state of the arm is not mentioned.

This was obviously a case of ulceration of the cartilages, originating in rheumatic inflammation, and very different from the case preceding it. The treatment, both of the disease of the articulation and of the phthisis, appears to us to have been rather too stimulating.

Some cases of other affections are related, but we must here conclude this notice. We have no doubt whatever, that the practice of the Infirmary will continue to be laid before the public by the able and zealous surgeons.

---

### II. Birmingham Infirmary for Diseases of the Eye.

A report of this institution, from March 1st to December 31st, 1832, has been published by Mr. Middlemore, assistant surgeon to the Infirmary, in the Provincial Transactions. The following statement of the gross number treated is useful, as affording a rough estimate of the relative frequency of various affections of the eye at Birmingham. The strumous affections will be found, as might be anticipated, to preponderate.

Simple acute conjunctivitis, 186. Chronic conjunctivitis, 79. Acute conjunctivitis, with pustules on the conjunctiva, 90. *(a)* Acute conjunctivitis, with pustule or ulcer of the cornea, 129. Acute conjunctivitis, with puriform secretion, 61. Purulent conjunctivitis of newly-born infants, 32. Irritable conjunctivitis, 24. Strumous conjunctivitis, 53. Pterygium, 5. Corneitis, 12. Vascularity of the cornea, 20. Opacity of the cornea, 99. *(b)* Conical cornea, 3. Staphyloma of the cornea, 13. Impaction of foreign bodies in the cornea, 13. Simple acute sclerotitis, 7. Rheumatic sclerotitis, 11. Staphyloma of the sclerotica, 3. Affections of the membrane of the aqueous humour, 10. Simple acute iritis, with or without ulcer or opacity of the cornea, onyx, or hypopion, 59. Chronic iritis, 16. Syphilitic iritis, 4. *(c)* Strumous iritis, 8. Prolapse of the iris, 8. Fungus from the iris, 5. Cataract, 17. Dislocation of the lens, 4. Choroiditis, 4. Retinitis, 3. Glaucoma, 5. *(d)* Hydrophthalmia, 2. *(e)* Amaurosis of various kinds and degrees, 63. Diseases of the lachrymal passages, 29. Epiphora, 7. Strabismus, 14. Tinea, 103. Lippitudo, 14. Hordeolum, 5. Ectropium, 2. Entropium, 7. Inflammation of the eyelids, 19. Œdema of the eyelids, 5. Nævus of the eye-lid, 3. Ptosis, 3. Adhesion of the eyelid to the globe, 4. Tumours in the eyelids, 11. Suppuration of the eye-ball, 7. Fungus of the eye-ball, 2. Wound of the eyeball and its appendages, 31. Disease of the bones of the orbit of various kinds, 2.

We fear that these muster-rolls are seldom accurate, being often confided to pupils who are not the best judges of some of the affections of the eye. We feel surprised that there occurred only twelve cases of corneitis, which, in London, is certainly a more frequent affection.

### Small-pox Pustule of the Cornea.

Mr. Middlemore remarks, that scarcely a week elapses in which he does not see one or more children whose vision has been more or less injured by small-pox. Children affected with small-pox are liable to the formation of a pustule in the cornea, which may accompany the development of the small-pox pustule in the skin, or in the mucous membrane, or may occur as the symptoms of the disease are subsiding in the cutaneous texture. Mr. M., therefore, recommends a careful attention to the state of the eye during the existence of small-pox, especially its earlier stages; for though it frequently happens that there is merely conjunctivitis, the disease of which mention has been made may take place. But to describe this affection:

It appears that the pustule forms, and is first witnessed, in the conjunctival covering of the cornea, and is then evinced as a small cloudy spot; the cornea becomes extensively inflamed; its interlamellar spaces are occupied by a rather glutinous matter. This matter is secreted in large quantity, and constitutes a densely opaque circular spot, of a greater or less extent; its pressure produces the absorption of the proper lamellar texture of the cornea, which, already weakened by the existence of inflammation, yields; and, in severe cases, the cornea ulcerates, and staphyloma is produced.

Mr. M. is in the habit of dropping the vinum opii into the eyes as soon as the pustule or pustules appear, and he thinks that this prevents the full development of the pustule.

### CONICAL CORNEA.

A young woman was sent to Mr. Middlemore with this distressing complaint. The right eye was perfectly healthy in appearance, and was *not* myopic. The cornea of the left eye was most beautifully transparent, and the projecting part of it had a dazzling crystalline appearance; its summit was slightly flattened, and apparently attenuated, but not at all opaque. The sight of this eye had gradually become dim and dazzling; when she looked with it alone, objects appeared multiplied. She had scarcely any pain or uneasiness of the globe. She had suffered for some time from a vesicular eruption on various parts of the body, but otherwise she was in health. She had never suffered from severe inflammation, or any injury of the eye, but she had done a good deal of fine sewing by candle-light.

The treatment adopted is the application of a blister twice a week, either to the temple, or above the eye-brow of the affected side—to use a weak solution of the nitrate of silver every other evening—to be careful in diet and regulate the bowels, and to rest the eye as much as possible.

Mr. M. observes that he has tried in one instance, and unsuccessfully, the plan recommended by Mr. Guthrie, viz. the exhibition of emetics and purgatives. He intends to give it a further trial, but he questions the propriety of exhibiting emetics in an advanced stage of the disease, where the cornea appears about to give way. He has found the introduction of a seton in the temple capable of arresting the disease in some instances.

### QUINA IN STRUMOUS AFFECTIONS OF THE EYE.

"I first pointed out, in the 'Midland Reporter,' the utility of the sulphate of quina in some cases of strumous inflammation of the cornea, the iris, and the membrane of the aqueous humour, occurring in connexion with certain constitutional symptoms. Soon after my remarks upon this subject appeared, the readers of the Medical Gazette were informed, by an anonymous writer, that the practice so advised had been previously suggested by Mr. Mackenzie, which, however, was not the fact. I have to repeat my thorough conviction of the propriety of the practice I formerly recommended, a conviction which subsequent experience has only tended to augment, although Mr. Guthrie, or one of his pupils, has stated (in the last number of the Medico-Chirurgical Review), in particular reference to my practice in such cases, that the adoption of similar measures has not been attended with much advantage at the Westminster Ophthalmic Infirmary. I have, however, in my possession, the voluntary testimony of many surgeons, in support of the paramount efficacy of the sulphate of quina, when suitably administered in judiciously selected cases of scrofulous inflammation of the cornea, the iris, and the membrane of the aqueous humour; and I mention this fact merely to justify the urgency of my recommendation of a practice, which I was the first to propose and adopt, and which has been singularly successful under the observation of those who are far more competent than I am, to ascertain and determine its merits."

To this we can only reply, that facts are facts. We do not doubt Mr. Middlemore's word, but we do not find that strumous ophthalmia is cured in London by quina, as Mr. Middlemore seems to cure it at Birmingham. Undoubtedly quina or tonics of any sort, and especially steel and sarsaparilla, to which quina is not to be compared, are useful in strumous affections of the eye as in strumous affections of other parts. But they must be given on general principles, that is, the same principles must regulate their employment in this as in other strumous complaints, and we firmly believe that they are not one whit more efficacious in one than in the other, in short, that quina has no specific or peculiar powers in strumous affections of the eye.

### Hydrophthalmia.

Sarah Miller had dropsy of the eye, apparently dependent on an increase both of the aqueous and vitreous humors. Various medicines and applications had been employed in vain. As there were great pain and tension of the globe, Mr. M. punctured the cornea, but with only temporary advantage. Mr. M. therefore removed a portion of cornea at its centre, about equal in size to the plane surface of a split-pea. The aqueous humor and lens were immediately forcibly injected through the opening, and the pain was at once relieved. After the wound had healed the pain returned in a slight degree, and Mr. M. fears that it will be necessary to repeat the operation. Mr. M. makes the following observations on the treatment of this complaint.

"I was aware, in this instance, of the great advantage of removing a large portion of the cornea, but I was also conscious of the objections to this more extensive operation. Sometimes the removal of a small portion of the cornea, (Celsus says, "*ad lenticulæ magnitudinem,*") is sufficient to cure the disease, and it is desirable to try its effect, at least, for I have known the extensive excision of the front part of the eye, when it has been much enlarged from hydrophthalmia, followed by very severe symptoms. The excision of the cornea merely destroys a portion of the secreting surface, yet, notwithstanding, it is often succeeded by a cure of the disease, (in as far as preventing the painful distention of the globe can be termed a cure) although the septa of the hyaloid membrane still continue, and retain, as I presume, their secreting power.

In recommending this operation, I am, of course, supposing cases in which the disease cannot be cured by the abstraction of blood, by the use of counter-irritation, and by the administration of medicines. The occurrence of dropsy of the eye-ball, in connexion with dropsy in other parts of the body, is so exceedingly rare, that many of the oldest and most experienced practitioners in this town, have assured me they have never seen the two diseases co-existent.

It will be understood that I do not recommend the excision of the cornea, until the effect of tapping the eye has been ascertained; but where that surgical measure has been fruitlessly adopted on several occasions, I should certainly think it preferable to remove a portion of the cornea, rather than tap the eye, as is sometimes done forty or fifty times, at occasional intervals.

After the cornea has been removed, the new matter which is deposited from its incised edges, is sometimes projected forward by the contents of the globe; this occurrence takes place before it has acquired sufficient firmness to resist the pressure from within. The result of this projection of the matter of reparation is, frequently, the production of large staphyloma; with a view of preventing this untoward occurrence, I have recommended that, as soon as the central portion of the cornea (which should be oblong in the lateral direction,) is excised, its divided edges should be brought together by means of a fine suture, which should not be removed until the parts are pretty firmly united, which may be expected to take place in four or five days. This practice has been successfully adopted, at my suggestion, upon the eye of a gentleman, who was affected with large staphyloma corneæ, and whose engagements would not allow him to remain at Birmingham, under my own care, whilst the operation for its cure was performed, and for a proper period afterwards."

### STRYCHNIA IN AMAUROSIS.

The present report concludes with some remarks on this head, and a case in illustration. Mr. M. observes that he has observed that, "although the propriety and great advantages of this practice are manifest and pretty generally acknowledged, many medical men appear strangely reluctant to adopt this mode of cure." We fear that the latter part of the sentence is more strictly accurate than the former; the reluctance is undoubted, the advantages of the method not so manifest nor so generally acknowledged as Mr. Middlemore believes. We have seen the strychnia tried in several cases of amaurosis, and we must candidly own that it did no good, at all events no permanent good in any. They were cases to which the treatment seemed applicable, but it failed. On the other hand, the strychnia produced unpleasant symptoms in two or three instances. We have heard professional men make similar remarks, and although we know that the practice has been tried in several institutions, and that every disposition existed to try it fairly and with a leaning in its favour, yet now it is seldom had recourse to, a pretty satisfactory proof that it has not answered. Mr. Middlemore, in reply, may appeal to his cases. As many or more have been brought forward in favour of the strychnine practice in general palsy, but what experienced physician has now much confidence in that practice, or rather has not an apprehension of it. Few will accuse us of being advocates of things that be because they are, or of old prejudices because they are old. But we cannot, as practical men, shut our eyes to the truth, that the flashy recommendations of new medicines have ended for the most part in complete disappointment. We do not hesitate to say that of late years our progress has not been in this direction, or effected by these means. Our substantial advancement has been through the medium of the facts of morbid anatomy, giving us a clearer insight into the nature of disease, and enabling us to determine with more precision what diseases are essentially structural and what are not. We have attained a more exact diagnosis, and have consequently applied with more precision and certainty the ordinary principles of treatment. Such we believe will be the acknowledgment of those whose experience has been sufficient to sober their views and their expectations.

We would not be unjust to Mr. Middlemore, nor have we any wish to dogmatise. Our opinion is, after all, but the opinion of an individual, and Mr. Middlemore may be right. We will give the case which he relates.

E. H., æt. 35, was so affected with amaurosis that he could only distinguish suddenly and extremely varying degrees of light. The eyes were dark coloured, the pupils large, the iris healthy but sluggish. He had worked, as a smith, nearly all his life at a bright fire. He was pale, by no means robust, and in a pretty good state of health. Having cleared out the bowels by a brisk purgative, Mr. M. placed a blister over each eye-brow, and sprinkled daily on the raw surface of each a sixth of a grain of strychnia, gradually increasing the quantity to two grains. In the course of a few weeks vision was "very much improved," but he became tired, and entered again on the infirmary books. The treatment was of no service to him, but he could see tolerably well for one or two hours during the day, and could find his way about the streets, without being led by his child as formerly. He now again employed the strychnia, and his sight underwent a marked and progressive amendment, but not being able to support the painfulness of the treatment, he again discontinued his attendance. He afterwards went to the hospital, where he remained for about six weeks, but derived no benefit. His sight is now sufficiently restored to enable him to follow the coarser parts of his business. Mr. Middlemore observes that this is "a result of the application of the strychnia."

---

### III. Bristol Infirmary.

#### Case of Osteo-Sarcoma, in which considerable Portions of the Upper and Lower Jaw, were removed.

This case is related in the Provincial Transactions by Mr. Hetling, the operator. As it is a matter of the highest importance that the profession should be as well acquainted as possible with the results of great operations, we shall lay the case before our readers.

*Case.* Margaret Hunt, æt. 23, sallow, of scrofulous habit and feeble intellect, was admitted into the Bristol Infirmary, Jan. 27, 1831, with a large tumour on the left side of the face. The tumour extended over both jaws, elevated the integuments of the cheek, and partly protruded between the lips at the angle of the mouth. On examining the interior of the mouth with the finger, it was ascertained that the tumour originated in the superior maxillary bone—that it had extended backwards into the palatine portion of the mouth—and was pressed forwards and downwards on the lower jaw by the action of the cheek, producing extensive absorption of that bone—that the alveolar border and processes of both jaws were nearly all removed from that side of the face—that in the upper jaw there was not a tooth left, and in the lower only a few, loose and buried in the medullary matter of the tumour. The tumour having insinuated itself between the jaws of course prevented the closure of the mouth; the cheek was not adherent to the surface of the tumour, circumstances so far favourable to an operation. The tumour was about the size of a large orange. Externally it felt firm from the extension of the cheek; internally, of the consistence of brain. The glands of the neck were free from the disease. The patient could not masticate and could scarcely articulate; she was compelled to live upon fluids, and even these were swallowed with difficulty.

The friends stated that the girl had always been of a weakly constitution—that the swelling had commenced from exposure to cold, when she was between eleven and twelve years of age—that the swelling had been gradual in its increase, attended with pain and a fetid discharge—and that latterly there had been frequent and copious bleedings from the mouth. Nothing of any consequence had ever been done for the complaint.

In consultation it was determined to remove the tumour, and to destroy, if possible, the surface of bone from which it originated. A few days previous to the operation Mr. Hetling took away the remaining teeth. He determined *not* to commence by tying the carotid.

"Feb. 16.—The patient was laid horizontally on the table, on the right side, the head moderately elevated on a firm pillow, so as to place the cheek in the most favourable position for *the operation.* Two fingers were introduced at the angle of the mouth, between the tumour and the cheek, in the direction of the articulation; the integuments were then freely slit up between them to the lobe of the ear; another incision was then carried from the infra-orbitary ridge across the centre of the first incision, down to the angle of the lower jaw. It was here necessary to tie some branches of the facial artery, which bled rather freely. The flaps of the crucial incision were then reflected, which fully exposed the external and irregular lobulated surface of the tumour, and afforded, also, the opportunity of tracing its base and attachments. The base of the tumour was found to occupy the palatine and maxillary portion of the upper jaw, and in its extensive growth its head had been forced down and attached to the ridge of the lower jaw, nearly as far as the symphysis, extending along the whole of the alveolar border nearly in a horizontal line from the mental foramen to the condyloid process, the whole of which portion was discovered to be either absorbed or in a state of caries from the long-continued pressure of the tumour. In fact, the tumour had so worked its way across the lower jaw, both inwards and outwards, that it was found buried in its substance, and, consequently, absorption of its body had been going on for some time, on both sides of the bone. The substance of the tumour was next separated by the knife and fingers, from its base and adhesions; this occupied a considerable time, in consequence of its various sinuations, as it had penetrated in every direction where it could find access. When this was effected, an extensive irregular surface of bone was found in a state of caries, extending, in the upper jaw, from the pharynx across the palate to the malar bone. Not the least vestige of the thin walls of the antrum remained. Fortunately the floor of the orbit was left uninjured. With the assistance of Liston's bone-cutter, small saws, &c. every portion of diseased bone was taken away that could be safely removed, and the general surface scraped, as carefully as possible, with the knife, it being intended, finally, to apply the actual cautery over the whole plane of the diseased bone. Having accomplished this tedious and difficult part of the operation, ample room was found for amputating the lower jaw at the articulation; caries having extended, as before stated, from near the symphysis along the whole of the upper margin to the joint. This extensive line of bone was then sawed off, except the condyloid process, which was afterwards easily disarticulated, and removed with Liston's bone-cutter, having first divided the fore part of its capsule, and also the temporal muscle from the coronoid process. This important and generally intricate part of the operation, was facilitated by the previous removal of all the teeth and alveolar processes."

The operation occupied about three quarters of an hour, and in consequence of the exhausted state of the patient, the actual cautery was not employed. The flaps of the cheek were brought into apposition by three sutures, adhesive straps, and a light bandage. On the fourth day the dressings were removed; the external incision had completely united with very little deformity. In a fortnight the dressings were discontinued, and the patient able to walk about the ward convalescent. In the course of a month she masticated as well as usual with the other side of her mouth, from which there was then no discharge. On the 5th April, seven weeks after the operation, she was dismissed apparently cured.

On examination of the tumor, its lobulated shape was in a great degree destroyed, in consequence of laceration with the finger in the operation. Its substance throughout possessed the common homogeneous structure of medullary matter. On cutting it into slices, a few blood-vessels were divided, from the orifices of which a little blood oozed out. In fact this was a case of medullary sarcoma.

It might be thought by some of our readers that this was a splendid instance of the powers of operative surgery. Now mark the result.

Some time after the patient left the infirmary the disease reappeared. She could not be prevailed upon to return to the hospital, and, after languishing for about a twelvemonth, she died.

We wish all narrators of operations were as anxious to ascertain results and as punctual in publishing them as Mr. Hetling. We should not then have so much to induce the profession to admire and practise operations for the extirpation of malignant tumors. When we see how seldom even amputation of a limb in which there is a fungoid tumor, of a breast or of a testicle affected with the disease, when we see in fact how seldom complete extirpation of the part is successful, we have little reason to expect that the mere dissecting away of such a tumor from the jaw, even though the actual cautery be subsequently liberally employed, will be unattended with failure. In a report from St. George's Hospital, a little farther on, our readers will find two other cases of malignant disease of the jaw, in which operations have been unsuccessfully performed.

IV. Liverpool North Dispensary.

Case of Melanosis.

So few circumstantial details of this singular form of malignant disease are presented to the public, that we are readily induced to draw attention to the following. It is narrated in the volume of Transactions to which we have already on several occasions referred, by Dr. Williams, Physician to the Liverpool North Dispensary, in which the patient was.

*Case.* John Thomas, a coal miner, healthy and sober, had on his right shoulder, near the base of the scapula, a purple or dark brown spot, a nævus, in short, about the size of a section of a pea. In 1826, he being then about 29 years of age, this spot was observed to increase. In March, 1827, the excrescence was as large as a marble, and was then frequently hurt in following his occupation. In December, a dark-coloured speck was observed in a line between the angle of the left side of the inferior maxillary bone and the left nostril; and, in a few days more, a similar one was noticed near the base of the right side of the lower-jaw. In 4 months or so after this, both specks began to spread, and others made their appearance from time to time on various parts of the body. In February, 1828, the excrescence on the back, which had assumed the mushroom form, began to discharge spontaneously and unceasingly a sanguineous fluid from its surface, which seemed excoriated. In April and May the excrescence was partially destroyed by caustic, but this did not stop its growth, though it did temporarily arrest the discharge, which, however, began anew in October, and in a few weeks became so troublesome that he left off work altogether. Soon after Christmas he went into the Manchester Infirmary, for the purpose of having the excrescence on the back removed, which was done by means of the ligature. The wound quickly healed. His health was now impaired. In a few days after his discharge from the Manchester Infirmary, he went to Liverpool, and on the 24th April, 1829, was admitted a patient of the Liverpool North Dispensary, for pain in the left side without cough. This was removed, and the patient now coming under Dr. Williams' observation, he carefully observed and has as carefully related the characters and progress of the melanotic affection.

At this time the melanotic depositions were pretty general on the surface of the body, but more especially on the scalp, face, and arms. They were characterized by the appearance of blackish specks, and of slightly elevated, flattish, globular, and conical-formed tubercles, with smooth shining surfaces, and of various sizes.

The blackish specks were found to be the initial forms of the sensibly raised spots, or flattish tubercles. These specks, when first visible to the eye, appeared to be deposited immediately under the cuticle, as if each one of them had occupied a pore of the skin. They spread themselves in a somewhat circular form, as if the natural tissue had been infiltrated with the melanose matter. No thickening of the part, occupied by these specks, could be appreciated before they had attained the diameter of at least three or four lines, but after attaining this growth, they, by degrees, put on the tubercular character, becoming hard, sensibly elevated, and their edges terminating, more or less, abruptly: a flattish tubercle on the forehead was elevated a line and a half, or two lines, above the adjacent parts. These tubercles were indolent, and spread slowly; they neither ulcerated nor softened, having, apparently, no natural termination, and when punctured, nothing seemingly escaped but a few drops of blood. None of these flattish tubercles were softened.

"The globular tubercles, in their initial state, presented themselves in small hard roundish tumours, which were evidently deeper seated than the initial specks of the flattish tubercles; for the former could be felt before they could be seen, whereas the latter could be seen before they could be felt. These roundish tumours, generally, were developed either in the cutis vera, or in the cellular membrane attached to its under surface, for they remained fixed and immoveable in one situation; a few however, from changing their situation, were evidently developed in the deeper-seated subcutaneous cellular substance. No discolouration of the integuments marked the site of any of the tumours, until they approximated the surface, so as to be pretty prominent; then their respective apices assumed a blackish hue (three or four had a sort of reddish yellow cast), and a smooth shining surface. These globular tubercles were indolent, insensible, and of a slow growth. The sizes which they individually attained were various, from that of a marrowfat pea to that of a pigeon's egg; some exceeding the latter size, and a few, in their progress, assumed the ovoid, or, rather, the teat-like form, and rose from half an inch, to an inch and a half above the adjacent surface. These globular and ovoid tubercles, when first developed, felt consistent and solid; afterwards this character was changed for that of fluidity.* These tubercles softened at all sizes. While on the increase they retained their primary hardness, but, as soon as they softened, they ceased to grow. As the teat-like tubercles, and the globular ones that were fairly developed above the surface of the skin, softened, they lost their smooth shining appearance, and shrivelled. This change took place from their contents being partially absorbed, or exuded, imperceptibly. I think the fluid was exuded, as scales were constantly peeling off their surfaces, but none peeled off the tubercles that retained their hardness; though the subcutaneous globular tubercles softened, yet no diminution took place in their bulk, which would have been the case, had their contents been absorbed. On puncturing the softened tubercles, black-coloured matter escaped."

Both kinds of tubercles continued to increase in number, until at last they occupied every part of the surface of the body except the penis, scrotum, and the ears. The colour, generally, was a mixture of the blackish purple with the blueish black. One subcutaneous tumour exhibited a blueish-grey tint. This tumour attained the size of a hen's egg. As the disease advanced, the skin in the interstices, between the tubercles, became gradually darker and darker, and a fortnight before the patient's death, his visage darkened very rapidly. Just before his death, the skin had all the appearance of being infiltrated with the melanose matter.

The cicatrix on the back continued sound till the beginning of August, when a blackish tumor sprang up from its centre, as did another from the surface of a flattish tubercle, situated a little below the cicatrization. These tumours were indolent, assumed as they grew the mushroom form, and discharged from their surfaces a thin, sanguineous fluid.

The effects on the general health may be shortly stated as the following. A pleuritic attack, which has been mentioned, and one on a previous occasion. After the pleuritic attack, a degree of debility, without sensible emaciation or apparent ill-health. Early in June, headache, which ever afterwards troubled him—for the last two months of his existence, to such an extent as to produce stupor. From the beginning of August, his health gave way rapidly; he lost flesh; his secretions became depraved; his urine was high-coloured, scanty, and deposited a sort of coffee-ground sediment, but was neither acid nor albuminous. The perspiration had a peculiarly rank smell, and, towards the latter end of his life, it was colliquative. A few weeks before his dissolution, his debility and emaciation were extreme, and for some days before his death, on the 15th Nov. 1829, he was comatose.

About the latter end of August, a tumour, that in time discharged a brownish-coloured fluid developed itself in the left antrum maxillare.

Towards the middle of October, a tu-

* "This circumstance favour the opinion entertained by M. Laennec and Mr. Fawdington, namely, that Melanosis has two stages, the first a stage of crudity, the second of *ramollissement*."

mour was developed in the epigastric region, rapidly extended to both the hypochondria, and finally attained an enormous size.

Unfortunately permission to examine the body could not be obtained.

It would be impertinent in us to point out in what particulars this disease resembled, and in what it differed from, medullary sarcoma. It is evident that the patient died of visceral disease, and it is probable that the latter was either melanoid or fungoid. The headaches and final coma make it probable, also, that similar disease attacked the encephalon or its envelopes, as occasionally happens in cases of medullary sarcoma. The profession are indebted to Dr. Williams for the minute description of the progress of the melanoid tubercles which he has presented.

---

### V. Bridgewater Infirmary.

#### Fixing the Scapula in Cases of Dislocation.

Mr. Toogood, senior surgeon of the Infirmary, has made some remarks on this subject. Mr. T. appears to be a man of few words, and whether his observations be or be not too good, they are certainly too short. To compress them, would be putting a flea in a rack. In cases of dislocation of the humerus, Mr. Toogood thinks that sufficient attention is not paid to fixing the scapula, and for many years past he has adopted a very simple method, which has never failed. It is this.

Having seated the patient on a low chair or stool, firmly secured the body and fixed the pulley, I stand over him, and place the heel of my right hand on the acromion, leaning my weight on my hand: by this means the scapula is fixed and rendered immoveable, the extension is then made, and the reduction quickly completed.

---

### VI. Rathkeale Dispensary.

Dr. Patterson has published, in the eighth Number of our esteemed Dublin contemporary, some "Observations in Dispensary Practice," some of which we will advert to. The first are on the—

#### Asphyxia of New-born Children.

On this head, we beg to direct attention to the remarks of M. Cruveilhier, an account of which is to be found in the Review department of our present No. The object of Dr. Patterson is to suggest the employment of cold affusion in these cases, and he cites two in support of the recommendation.

*Case* 1. "Mary O'Brien of Rathkeale, aged 24, in June, 1831, was seized with premature labour of her first child. She stated, she had only reached the commencement of the eighth month of gestation, and attributed her impending abortion to fright. Her pains being slow, after about thirty hours of lingering labour, she was delivered of a still male infant. Immediately on the protrusion of the fœtus, a very languid circulation was perceptible in the funis, and a slight motion of the limbs was observed. Previous to the division of the cord, it was attempted to excite respiration; but the funicular pulse having quickly ceased, the child was removed and subjected to artificial inflation, friction, external warmth, and nasal irritation. There being no appearance of benefit, and ten minutes having been lost in these fruitless attempts, I placed the infant in a tub, and twice dashed over it three quarts of water, the temperature of which was about sixty degrees. On the first dash, a slight convulsive motion of the body was sensibly excited; after the second, the heart and lungs were in evident action, but this was exceedingly weak and tremulous. While the babe was allowed to remain for a few moments in the water, which scarcely reached its ears, the thoracic parietes were subjected to strong friction. In effecting this, the integuments were made to glide, to and fro, over the ribs, so as to excite titillation. Movements of the arms and legs, and active respiration having quickly succeeded, the child was removed from the vessel, well dried, and wrapt up in flannel. It slowly acquired strength and activity, but ultimately, became remarkable for its large size and healthy appearance."

In the second case, there was no appearance of life, yet vitality could not have been long extinct. The funicular connexion was, therefore, speedily separated, and immediate recourse had to cold affusion. At first a momentary shuddering was observed, and, in the next instant, the heart's action was comparatively vigorous. The child recovered, and both are now living.

### Hemeralopia.

This disease is not very unfrequent in the Doctor's part of Ireland, and it has appeared to him that it has arisen from visceral derangement, and not from solar or other external influence.

*Case.* James Dwyer, aged 19 years, applied at the Rathkeale Infirmary, the 9th September, 1831. He complained that every evening at dusk, he became almost blind, and continued so till sunrise next morning; that he could see very imperfectly by fire or candle light, and that exposure to heat and active exercise, or labour, always considerably aggravated his inability to discern objects at night. He stated, that in candle light, however vivid, he could distinguish no object that was directly opposite to him. He could see a little in an oblique direction, and this enabled him, with much difficulty, to grope his way. The pupils were permanently and very much dilated. 'He felt stupid and confused in his head.' There was great fulness, hardness, and some tenderness of the right hypochondrium; conjunctiva yellowish; alvine evacuations white. Early in May, 1831, he was attacked with jaundice. He did not get rid of it for a month, and while labouring under the disease, he first noticed the derangement of sight. This continued, with only daily intermissions, up to the period of his application.

Having been bled, blistered, and purged, with little effect, he was directed to rub in one drachm of strong mercurial ointment every night, and as soon as this had affected the constitution, recourse was had to the nitro-muriatic acid bath. The moment the gums became tender, he experienced a manifest improvement of his sight. But at this time he complained, for a few days, that exposure to artificial light produced most unpleasant sensations in his eyes. Under the treatment, the hepatic disorder was quickly and effectually relieved, and with it the amaurotic affection entirely disappeared

Two other cases are related; but symptoms nearly similar were relieved by a nearly similar treatment. In all the cases there was, or there had been, jaundice, and, in several, the nitro-muriatic acid bath was sufficient, without mercurial action, to produce a permanent cure. In every case of jaundice, Dr. Patterson has observed a tendency to hemeralopia. We fear that, if the bent of an enquirer's mind were towards the existence of this affection, he could easily, by the questions he put, obtain from an amaurotic patient a sort of history of hemeralopia.

### Iodine for Caries of the Vertebræ.

The benefits of iodine seem something like the philosopher's stone—ever on the point of being discovered, and ever evading the searcher. We have daily accounts of the virtues of this substance —we hear that it is useful in bronchocele, in malignant tumours, in scrofula, in secondary venereal symptoms; yet, somehow or other, it has so happened that we have rarely seen it of much service in any of these affections. Still we must report the powers it possesses in the hands of others, and we hope that our readers will find them as great when invoked by themselves.

Dr. Patterson relates three cases of caries of the vertebræ, in which he employed iodine with much apparent effect, and he concludes by saying, that from the result of those cases, he has little doubt that iodine will be found a remedy no less efficacious in the treatment of this formidable disease than it has proved in bronchocele.

*Case* 1. In Aug. 1831, Dr. P. found J. Moran, æt. 14, an out-patient of the dispensary, suffering under extensive disease of the dorsal vertebræ, the three middle bones of which were principally engaged. He was unable to walk, and could only attain the semi-erect posture by grasping his hands with his thighs. The body of the sixth vertebra seemed totally destroyed, that of the fifth and seventh nearly so. There was unremitting and severe pain in the back. His countenance was pale, the body and limbs wasted, and there was troublesome diarrhœa, alternating with profuse night-sweats. Two issues, which had been open for nine months, existed on the back. On the 24th, having previously tried ineffectually quinine, cicuta, hyosciamus, Dr. P. ordered the lad five drops of the tincture of iodine every eight hours. On the 17th October, it is reported that all pain and uneasiness, save those resulting from the issues, had ceased; the issues were directed to be healed. On the 31st, Dr. P. saw him again. He had acquired flesh and the aspect of health, walked firmly without assistance, had experienced no pain since the removal of the issues, the diarrhœa and night sweats had ceased, and the functions were natural. Dr. P. has since heard that he enjoys good health.

*Case* 2. Mary G., æt. 26, became a patient, Nov. 12th, 1831. She principally complained of pain across the loins, where a slight projection of the spinous processes of the last dorsal and first and second lumbar vertebræ was observed; pressure here produced pain shooting down the right thigh. An abscess discharged profusely in the right groin—she had numbness and weakness of the inferior extremities—bad appetite—irregular hectic symptoms. She had been ailing for several months. Five drops of the tincture were given thrice daily, and the dose raised to ten drops, which were never exceeded. She improved rapidly in health, the inguinal discharge gradually ceased, and in three months her recovery was so complete that she discontinued the medicine.

*Case* 3. In July, 1832, Dr. P. was requested to visit Miss ——, æt. 14, of delicate appearance. Four or five years previously she had suffered from severe pain in the back with loss of power in the lower extremities, for which two issues were employed for twelve months, when the young lady was thought cured. She had remained well till the preceding April when she again began to suffer from pain in the back, her health suffered, she suffered from numbness and shooting pains in the thighs, and inability to direct the feet. On examining the back, the spinous processes of the four lower dorsal vertebræ were found to project about the fourth of an inch beyond their fellows, the projection not having the aspect of that produced by destruction of the bones. Pressure here occasioned much pain, felt chiefly in the situation of the eleventh and twelfth dorsal.

Tartar-emetic ointment was applied to each side of the spine, and tincture of iodine administered in five-drop doses every eight hours. In three weeks all pain of the back and extremities had ceased, but, a copious epistaxis occurring, the iodine was suspended. The hæmorrhage ceased, and in four or five days the vertebral pain was as bad as ever. The medicine was resumed, and the pains "almost immediately" disappeared, the health became greatly improved, and the young lady, now, Nov. 1832, walks erect with ease and firmness.

None of the preceding patients observed the horizontal position. We hope Dr. Patterson's sanguine anticipations may be realized, and that iodine may display some powers in cases of caries of the vertebræ. We are sorry to say that we have our doubts, for medicines that the profession find comparatively powerless have been ushered into notice with cases quite as striking as these.

---

### VII. Bristol Infirmary.

### How a Case of Inguinal Aneurism should not be reported.

There are two methods of conveying information; one is by setting a good example, the other by setting a decidedly bad one. Thus a pupil will frequently learn as much by witnessing a very bungling operation, as a very adroit one. A gentleman, who is too diffident to sign his name, has contrived to make a most instructive report in the Lancet for June 15th. We defy any surgeon, pupil, or apprentice to concoct one more calculated in every respect to shew what should be avoided. It is a model of deformity. We should be sorry that a specimen of such value should not be presented to our readers, and we hasten to transfer it to our pages for the purpose of pointing out its various excellencies.

" Residing in the neighbourhood of Bristol, and learning lately that there was a case of inguinal aneurism in the above institution, I requested permission to see the operation. It was granted immediately. We waited somewhat impatiently about an hour, when Mr. Richard Smith, the senior surgeon, came into the room and said, 'Gentlemen, I am sorry to inform you that I cannot persuade the man to submit to the operation, although he had distinctly consented to it; but if I can hereafter alter his resolution, I shall be happy to see you all again.' We departed, much disappointed. A week elapsed, and on the 21st we again assembled. In a few minutes the patient was brought in, seemingly care-worn and woe-begone. He was aged about 40, and dated his malady to a kick from a rioter, when the prison was burned in October, 1831, he being a turnkey.

The perforation in the artery was, probably, nearly about under the centre of Poupart's ligament, extending in a circle up the abdomen and down the thigh, about two inches and a half each way. It pulsated very strongly over the whole surface, and every stroke was visible in the gallery.

This unfavourable extension of the disease had rapidly increased during the week which had elapsed. It obliged Mr. Smith to begin his incision higher than is usual. The operator drew his scalpel at one sweep entirely through the integuments in a somewhat semilunar direction, making a wound about five inches long, and ending at the upper edge of the tumour. The external oblique was thus completely exposed. The fascia was next lightly cut through, and a director was passed down, upon which the muscle was slit to correspond with the external opening of the integuments. The operator now passed his left index from across, and entirely under, both the internal oblique and the transversalis, ran the back of a Pott's knife along his finger, divided the whole, and thus exposed a considerable surface of the white peritoneum. Laying aside his knife, the way was cleared with both hands down to the psoas muscle, and the operator began to search the cavity. One of the surgeons who was near, asked 'Do you feel the artery?' 'Yes, distinctly, and it is a good deal bound down, but I think I shall soon be able to detach it with my finger and thumb.' Presently Mr. Smith said, 'Now I have the artery over my finger.' 'Is it quite free?' 'Yes, there is nothing but the artery; I am quite sure of it; favour me with the blunt needle with the ligature.' The instrument broke from the handle, another was substituted, and the needle was passed very readily under the vessel. A surgeon pulled up the ligature with a dissecting forceps, and the needle was then withdrawn. The silk was now under the artery. The operator drew up the ends so as to bring the sides of the artery in contact. 'Gentlemen, will you satisfy yourselves that the circulation is commanded?' Three or four hands in succession were laid upon the tumour. 'Yes, the pulsation is gone—it is all right—the sooner you tie the better.' The ligature was then tightened, and the operation finished. The integuments were drawn together with adhesive plaster, a strip of lint and bandage were applied, and the patient was carried out. The operation occupied under ten minutes, and there was not a tablespoonful of blood spilled from first to last. After the first incision, the patient appeared scarcely to suffer at all. The operation gave universal satisfaction. It was performed with the utmost coolness, and in a surgeon-like manner. I have seen

Sir Astley Cooper perform the operation twice, and I have seen three other hospital surgeons perform it in London, but this operation in my judgment might dispute the palm with any of them. The utmost attention also was evinced by the other four surgeons, Messrs. Hetling, Lowe, Daniel, and N. Smith, and the duties of Mr. Morgan, the house-surgeon, were performed with promptness.

Five days have now elapsed, and I understand that the man has had not one unpleasant symptom; he has slept well; there has been no tension of the abdomen, the wound exhibits a mere line, the tumour sinks, and the limb is of a comfortable temperature. This case is the second of the kind which has occurred in the house.

Mr. Smith has been surgeon to the institution for nearly forty years."

For a fact to be well recorded two conditions are necessary; clearness and accuracy of description, and the exclusion of irrelevant particulars. When these conditions are observed, and a decent respect evinced for grammatical construction, a case may be considered as well reported. Let us see how this nameless gentleman succeeds. And first of his grammatical peculiarities. "The operator," says he, "now passed his left index *from across*, and entirely under both the internal oblique and the transversalis, ran the back of a Pott's knife along his finger, divided the whole and thus exposed a considerable surface of the white peritoneum." Setting aside the utter impossibility of understanding what is meant by passing an index from across and under a muscle, and the obliging manner in which the white peritoneum is introduced, in contradistinction we suppose to a peritoneum of some other colour, we beg to suggest, that if the ordinary principles of construction are to guide us in the perusal of medical reports, Mr. Smith divided his *finger*, as well as the patient's muscles, in the most gallant and unhesitating manner.

Let us look at the clearness of the descriptive portion of this interesting communication. "The perforation in the artery was, probably, nearly about the centre of Poupart's ligament, extending in a circle up the abdomen and down the thigh, about two inches and a half each way. It pulsated very strongly over the whole surface, and every stroke was visible in the gallery." This is certainly an extraordinary case, and any journal, weekly, monthly, or quarterly, would naturally be anxious to publish it. Think of a *perforation* of an artery extending in a *circle* up the abdomen and down the thigh, two inches and a half each way. But this is not all. This "down the middle and up again" perforation, pulsated, and every stroke (of the perforation) was visible in the gallery. What the gods must have thought of it we do not know, but we conceive that the reporter has very wisely put the whole affair as a probability only. To be sure there are difficulties even in admitting this, and we would suggest that the unsuspecting gentleman meant tumor, when he talked of perforation. We fear, however, that we must abandon this suggestion, as the very next sentence informs us that "this unfavourable *extension* of the disease had rapidly increased during the week which had elapsed," so that the perforation turns out after all to be an extension.

The description of the commencement of the operation reminds us of the manner in which Smollett makes Beau Didapper draw his hanger; but how Mr. Smith disposed his incision is, from the said description, a delightful mystery.

We have observed that one feature of a well-recorded fact is the absence of irrelevant circumstances. How well our reporter has displayed this is shewn in the anxious dialogue so dramatically related. We know of nothing possessed of such intense interest, with the exception perhaps of the duet between the sisters in a pathetic drama denominated Blue-beard, or Female Curiosity, or the dialogue between Beefington and Puddingfield, in Canning's Play of the Rovers, or the Double Arrangement.

"Yes, the pulsation is gone—it is all right—the sooner you tie the better."

"PUDDINGFIELD.—*(With extreme earnestness.)* Its name?

BEEFINGTON.—The Daily Advertiser.—

PUDDINGFIELD.—Oh ecstasy!

BEEFINGTON.—*(With a dignified severity.)*—Puddingfield calm yourself—repress those transports — remember that you are a man." &c. &c. &c.

There is one other circumstance worthy of remark in this report; it is the extreme good nature of the reporter. Every thing is praised—operator —assistants — house-surgeon, and all the inanimate things, gallery, museum, and so forth. It were much to be wished that more of this spirit existed, and we should not be deemed so pugnacious a profession.

Seriously, we have noticed this case because we think it disgraceful to the medical press to promulgate such crudities. Is it reduced to such a state that any communication, the most illiterate, is acceptable; or does it reserve its criticism for books, and display in such communications as these, all the vices of literary omission and commission, that it reprehends? We cannot believe this, and we do hope that the editors will exercise a more rigorous scrutiny into the matter and the manner of the articles they insert. Such a case as that we have now noticed can do no possible good to science or to any thing else, and can only bring periodical literature into contempt. It may readily be believed that we entertain no personal feeling in this case, being utterly ignorant of the name of the author.

---

### VIII. COUNTY OF DUBLIN INFIRMARY.

### MR. CRAMPTON ON THE PATHOLOGY OF DISLOCATION OF THE SHOULDER-JOINT.*

If there is any thing which has afforded us greater pleasure than the establishment of a Medical Journal in Dublin, it has been the distinguished manner in which that Journal has been conducted. For general soundness, acuteness, and manliness of criticism, it has not been excelled, and we feel delighted at meeting what we may, perhaps with some vanity, denominate, a worthy fellow-labourer in the field of criticism. The Dublin Journal and this Review stood alone in the exposure of the absurdities and fallacies of Dr. Stevens, and while our contemporary displayed the weakness of his chemical theories and experiments, we ridiculed the general tenor of the work. Both of us were exposed to some little obloquy at the time, but we venture to say, that there are few who now think of Dr. Stevens' saline treatment but as a folly past, if they think of it at all. The Quarterly Review, indeed, has thrown its mantle over him, but, like the mantles of the Senators on Romulus, it has crushed its wearer, and his spirit is gone from us, none know whither.

The Dublin Journal has been as distinguished in its original as in its critical department, and we have shaken hands with Crampton, and Stokes, and Graves, and others of the Lords of the Emerald Isle. Late, say we, may be the parting, as the greeting has been pleasant and hearty.

The object of the present article is to introduce to the notice of our readers some observations of Mr. Crampton's on Dislocation of the Shoulder-joint. Though not strictly a hospital report, it has so much of the clinical about it, so genuine a smack of the hospital still, that we cannot introduce it in a more appropriate place than this. Mr. Crampton will appreciate our feelings when we say, that we never see a bird of his rise from cover, so blithe and so strong on the wing, but we like to bring it down.

Mr. Crampton justly observes that though the *treatment* of dislocation of the shoulder-joint has engaged much attention, yet a rational method can be founded only on an exact knowledge of the pathology of the injury, which, unfortunately, has been but sparingly illustrated. It is Mr. Crampton's wish to add to this knowledge. At the time

---

* Dublin Journal, Nos. VII. and VIII.

Mr. Hey wrote, Mr. Crampton is not aware of there being more than one case of dissection of a shoulder-joint recently dislocated, on record, that of Mr. Thompson's in the Medical Observations and Inquiries for 1761. Even in this case the description is confused, and the accompanying plate imperfect. The case, too, was not, strictly speaking, a recent one, eighteen days having elapsed between the reception of the injury and the examination of the joint after death. In Professor Bonn's monograph on Luxations of the Shoulder, the most recent case of which a dissection is given, is of two years' standing. This monograph was published in 1782. In Sir Astley Cooper's Treatise on Dislocations, we are presented with the post-mortem examination of two cases of recent dislocation of the humerus, both of which were instances of luxation "into the axilla."

In these cases we are told that, in the first, the capsular ligament was torn on the whole length of the inner side of the glenoid cavity, which (rent) would have admitted of a much larger body than the head of the os humeri through the opening. The tendon of the subscapularis muscle was also extensively torn, but the tubercle on which the supra and infra spinatus, and the teres minor muscles are inserted, was not, (as in Mr. Thompson's case,) torn off. In the second case, in which dislocation had existed (unreduced) for five weeks, the capsular ligament had given way in the axilla between the teres minor and subscapularis muscles, the tendon of the subscapularis was torn through, at its insertion, all the articular muscles, but particularly the supra-spinatus had been more or less lacerated, as it would seem, in the attempts which had been made at reduction. Sir A. Cooper found that "the resistance to reduction (even after death,) was such as he could not by himself overcome; he divided one muscle after another, cutting through the coraco brachialis, teres, major and minor supra-spinatus muscles, but still the opposition to his efforts remained; he next divided the deltoid muscle, and found that the supra-spinatus muscle was his great opponent, *until he drew the arm directly* upwards, when the head of the bone glided into the glenoid cavity."

To these cases Mr. Crampton is enabled to add two others which have fallen under his own observation: one of a recent dislocation downwards, and one of a recent dislocation under the pectoral muscle.

*Case* 1. In 1808, a labouring man was engaged in digging under the foundation of a house, when a wall fell upon him. He was brought into the County of Dublin Infirmary, in a dying state, in consequence of injury of the head. He lived only two hours. On examination of the body 18 hours after death, the right humerus was found to be dislocated into the axilla. Previous to reducing the dislocation Mr. C. made a careful dissection of the joint. The following were the appearances.

On removing the integuments of the axilla, the cellular membrane, which was extensively ecchymosed, formed a kind of cap, closely embracing the head of the os humeri, which when the axilla was cleared, was seen lodged on the inferior costa of the scapula, or rather on its neck; the head of the bone, in escaping from its socket, had pushed the teres minor downwards, and burst through the lower part of the subscapularis muscle, some of the fibres of which closely embraced the neck of the bone, while the bulk of the muscle was pushed upwards and detached from the inner surface of the scapula. The neck of the humerus, therefore, was in some degree embraced by the divided fibres of the subscapularis muscle, while a portion of its head rested on the neck and part of the venter of the scapula without the intervention of any muscular substance. The short head of the biceps and the coraco-brachialis were forced to describe a curve outwards over the neck of the humerus on the sternal side, while the long head of the triceps crossed the neck of the bone obliquely on the dorsal side; this strangulation of the head of the bone, by the surrounding muscles, was made most apparent when extension was applied to the fore-arm. The biceps and triceps seemed then to close behind the head of the bone, and interpose themselves between it and the glenoid cavity; the tendon of the long head of the biceps remained in its groove, but the sheath in which it runs was partially ripped up.

The capsular ligament was completely torn from the lower part of the neck of the humerus to the extent of more than half its circumference, the torn edge appearing like a crest over the head of the bone. The great nerves and blood vessels of the arm were forced to describe a curve backwards, by the pressure of the head of the bone which was in contact with them. The tendons of the supra-spinatus, infra-spinatus, and teres minor were completely torn from the humerus, carrying with them a scale of bone from the tubercle.

To ascertain the obstacles opposed to reduction, the scapula was fixed, the arm raised, and extension applied to the wrist. So long as the hand was held *supine*, the head of the bone remained immoveable, apparently from the closing of the biceps and the triceps behind it. On turning the hand *prone*, rotating the limb inwards, and continuing the extension, reduction was easily effected.

The appearances in this case differed from those observed by Sir Astley Cooper, and agreed with those related by Mr. Thompson. In the case of the latter gentleman, the head of the bone was lodged on the inside of the neck of the scapula, between the subscapularis and teres major—the capsular ligament was completely torn from the whole circumference of the humerus—the attachments of the tendons of the supra-spinatus and infra-spinatus were torn off, with the part of the bone they were inserted into—and some fibres of the subscapularis encircled the neck of the bone. In Sir Astley Cooper's case, on the contrary, the tendon of the subscapularis was torn through, but the supra-spinatus and infra-spinatus remained attached to the tubercle, and reduction could only be effected by relaxing these muscles. Mr. Crampton observes very justly that the comparison of these facts is sufficient to shew, that in apparently similar dislocations of the humerus there may be very different degrees of lesion, and consequently different causes of resistance to reduction.

*Case* 2. J. W. æt. 30, was precipitated twice consecutively into a burning lime-kiln, from a height of about 15 feet. He was carried to the Meath Hospital. In addition to severe burns and lacerations, there was a dislocation of the humerus under the pectoral muscle, which Mr. M'Namara himself reduced, by merely drawing the arm gently forwards and downwards with one hand, while he pushed the head of the bone towards the glenoid cavity with the other. The man died in the course of the day, and 18 hours after death the joint was dissected.

The dislocation was unattended with the rupture of any muscle, or the separation of any tendon from its insertion into the bone: by a slight effort the dislocation was reproduced, and the pectoral muscles being removed, the polished head of the bone was now seen lodged on the cervix of the scapula, at the root of the coracoid process, but extending nearly as far as the notch in the superior costa; it had passed out through a rent in the capsular ligament, *over* the *upper edge* of the tendon of the subscapularis, detaching this muscle from its connexion (which at this point is but slight) with the inner face of the scapula, and pushing its fibres downwards, so that they formed a curve which partly embraced the neck of the humerus; the supra and infra-spinatus muscles were on the stretch, but had suffered no injury. The cellular substance covering their tendons was deeply ecchymosed, so as to mark their course most distinctly. On replacing the head of the bone, the opening in the capsular ligament, through which it had escaped from its socket, could be distinctly seen. It was formed by a separation of the ligament from the interior side of the brim of the glenoid cavity from top to bottom; it was bounded at the top by the tendon of the supra-spinatus, and at the bottom by the inferior edge of the tendon of the subscapularis, the rent was continued as far as the root of the lesser tubercle of the os humeri, and was of sufficient extent, but no more, to permit the head of the bone to pass easily through it; the *inferior part of the capsular ligament, however, (the part corresponding with the axilla), was perfect.*

The great blood vessels and nerves lay to the sternal side of the head of the humerus, and were forced a little out of their course. The axis of the head of the bone, in its dislocated position, was scarcely a quarter of an inch higher than the axis of the glenoid cavity.

Mr. Crampton appends to these interesting and valuable facts some practical observations. We will endeavour to give their substance.

I. Resistance to the reduction of a recently dislocated shoulder would seem to be owing to spasmodic contraction of the muscles, and *not* "from the neck of the bone being tightly embraced by the ruptured capsular ligament." Dissection does not prove the latter, and therefore it is mere supposition. Muscular contraction then being the obstacle, we must diminish its power by bleeding, the warm bath, antimony, long-continued extension. Another excellent means of obviating muscular opsition is taking the muscles by *surprise.*

"If before assistants are called in, or any apparatus is applied, the surgeon, while he appears to be occupied merely in ascertaining the nature of the injury, applies a gentle extension at the wrist, and slowly raising the arm to nearly a horizontal position, suddenly pulls it upwards and a little forwards, (that is towards the patient's face,) while at the same time he as suddenly pushes the trunk backwards by pressing, with the left hand, below the axilla, he will in a great number of *recent cases* succeed by this simple process in reducing the dislocation. His success, however, will in a great measure depend on the *unexpectedness* of the attempt; he should, therefore, endeavour to divert the patient's attention from his proceedings, and I know of no means so effectual for this purpose, as inducing him to describe circumstantially every thing connected with the occurrence of the accident; this is a theme on which all patients, who are at all able to express themselves, are sure to expatiate with the greatest satisfaction, and once engaged on so engrossing a topic, it will require but a small degree of tact on the part of the Surgeon to seize the favourable moment when he can apply his force with the greatest advantage."

II. In luxation into the axilla, muscular contraction seems to operate chiefly by pressing the head of the humerus against the inferior part of the brim of the glenoid cavity; in Sir Astley Cooper's case this was mainly effected by the supra-spinatus. The obvious deduction is, to raise the arm to nearly a right angle with the body previous to extension, and not to use any force in pressing the head of the humerus upwards, as that must lock it more firmly.

III. The *direction* of the extension is now generally that of a right angle with the body. In the reduction by the heel in the axilla the direction is nearly parallel with the body, but Mr. C. thinks that more force is necessary with this method. Mr. White, of Manchester, drew the limb directly upwards, but the practice has fallen into disuse—a presumptive proof of its not possessing any superior efficacy. The method has lately been brought forward at the Hôtel Dieu as a novelty. We are not surprised at this. M. Dupuytren has no love for English surgery, and candour forms no part of his merits or acquirements.

IV. Extension is now pretty generally applied to the wrist in preference to the arm. It is less painful, if not more effectual.

"V. Great stress is laid by most surgeons on the advantage of fixing the scapula, as it is called; it may be doubted, however, whether the thing be possible, or if possible, advantageous. It is quite plain that a split cloth, or a napkin with a hole through which the arm is passed, can, when the arm is strongly extended, act only on the inferior costa of the scapula, or rather on the walls of the axilla formed by the edges of the latissimus dorsi, teres major, and pectoralis major muscles; the whole effect of this force can be no other than to push the inferior angle of the scapula backwards and upwards, consequently to direct its superior angle, and the glenoid cavity downwards, and, by acting on the pectoralis major and latissimus dorsi, to draw the head of the humerus inwards towards the ribs, that is, to remove it *from* the glenoid cavity. To obviate this objection, some surgeons recommend pressure to be made by the hand of an assistant on the acromion of the scapula, so as to push it backwards while the humerus is drawn downwards and outwards; but it is plain that unless the force which the surgeon applies to the head of the scapula to keep it back, be at least equal to the extending force which is applied to the arm, the scapula cannot be *fixed*, it *must* follow the arm; besides when the arm is raised, the deltoid fills up the sub-acromial space and renders it impossible to apply any appreciable force to the acromion. As the neck of the scapula cannot be pushed *upwards*, it it proposed by Bonn, to disengage the bones by pressing the head of the humerus *downwards*, at the moment when the extension is at the utmost; the proposal is a most rational one, and has been adopted for several years past in the County of Dublin Infirmary, as I think, with considerable advantage."*

VI. When a considerable power of extension is required, Mr. Crampton prefers a ladder to the pullies. One end of the ladder is fixed—the patient stands between the bars near this end—the wrist of the affected limb is tied by a jack-towel or handkerchief to the sides of the free end of the ladder at a convenient distance from the body—and thus a powerful lever is obtained, one end of the ladder fixed on the ground being the fulcrum, the other end being free, and its depression, by an assistant producing the extension. Counter-extension is, of course, effected by a towel under or in the axilla.

VII. It has been much mooted whether dislocation forwards is a *primitive* dislocation, and it has been supposed that every luxation is primarily into the axilla. The case related by Mr. Crampton seems to prove that it is, or at least in that instance it was a primitive dislocation, and as the inferior part of the capsule was not ruptured, it would of course have been injurious to have first reduced the head into the axilla. In such a case the clear indication is to force the head of the bone *backwards* towards the glenoid cavity, the axis of which is as nearly as possible in a line with that of the head of the humerus; this can be effectually done by applying a fulcrum immediately below the axilla, and using the dislocated arm as a lever of the first kind; the surgeon should therefore place his left arm extended horizontally, immediately below the walls of the axilla, between the dislocated arm and the chest, and then grasping the wrist in his right hand, he should draw the arm forcibly across the patient's body.

*Case.* The Hon. Colonel Gore was overturned in his carriage, and suffered a dislocation of the left humerus forwards. Mr. Crampton saw him in less than an hour after the accident. Standing before him, he placed his left arm extended horizontally under his axilla, and grasping the wrist in his right hand, he drew his arm rapidly across his body, so as to bring the hand in contact with the right hip; the bone snapped into the socket at the first effort.

Several lithographic drawings illustrative of the descriptions are appended to the memoir. It is altogether one of a very interesting character, and well worthy the attention of practical surgeons.

### IX. ST. GEORGE'S HOSPITAL.

### I. TWO CASES OF MORBID GROWTH FROM THE LOWER JAW, FOR WHICH AN OPERATION WAS PERFORMED.

*Case* 1. Edward Roberts, æt. 57, admitted December 21st, 1831, under the care of Mr. Brodie.

*March* 1. The condition of this patient at present is as follows.

* Contrast this with Mr. Toogood's brief remarks on fixing the scapula.

On the right side of the lower jaw, on its upper edge and inner surface, and on the contiguous portion of the inside of the cheek, is an ulcerated fungus, about the size of a crown-piece, or not quite so large. The edges are prominent, rather everted, a line or a line and a half above the surrounding surface; the ulceration cup-like, deepest in the centre, its surface pretty smooth, with rather large and florid granulations. The diseased part feels hard and callous, and extends from about the situation of the canine tooth to the ascending ramus of the jaw. Some thickening of the cheek immediately around the disease, and the jaw seems somewhat increased in thickness; bone not exposed. Externally, when the mouth is closed a prominence is observed on the cheek immediately below the malar bone, and corresponding in situation with the morbid growth within. Ulcerated part little tender on pressure. Complains of slight pain occasionally shooting to the ear and lower margin of the orbit. A slightly enlarged lymphatic gland felt immediately beneath the angle of the jaw.

Aspect pallid and unhealthy—some emaciation. Complains of cough, to which he is subject every Winter—expansion of chest imperfect—too great resonance of voice under clavicles, but not actual pectoriloquism. Action of heart rather strong—pulse full. Tongue moist—bowels irregular.

Says that he first discovered a lump in the site of the present disease in May or June last; he thought it was a gum-boil. The disease increased gradually, unattended with pain, and he is not aware when ulceration took place. Several teeth have been extracted at various times, so that at present none exist on the affected side posterior to the outer incisor. The disease has made progress since his admission.

On his entrance into the hospital he was ordered a dose or two of blue-pill and black-draught, which deranged his health a good deal. He afterwards suffered much from the cough, was considered to have phthisis, was turned over to Dr. Hewett, and after a certain length of time returned to Mr. Brodie. Dr. Hewett, we believe, had employed iodine in some form. The operation, which had long been postponed in consequence of the thoracic complaint, was performed on the 5th March, by Mr. Brodie.

An incision begun at the angle of the mouth and carried backwards through the whole substance of the cheek to opposite the angle of the jaw; another incision begun and ended at the same points, but inclosing with the last an elliptical portion of integuments, &c. Lower flap dissected downwards from the anterior edge of ramus of jaw to symphysis, exposing the external surface of the bone. Saw then carried through the mental portion, between the two left incisors. Right portion of jaw raised and directed outwards, and knife carried close behind it, so as to dissect it from its connexions within the mouth and towards the neck. Upper flap dissected from ascending ramus of jaw above the diseased part. Ascending ramus sawed obliquely from before backwards and downwards, and loose portions of jaw, &c. removed by a little dissection. Mucous membrane at commissure of upper and lower jaws being involved in the disease inferiorly, was dissected out. During the operation not many vessels of size required ligature, and no great quantity of blood was lost. One or two arteries bled smartly on the inner side of the sawed end of the bone at the ramus. The flaps of integument were brought together by several sutures, sticking-plaster, compress, and half-handkerchief.

The patient was now removed to bed, and soon afterwards bleeding occurred, and about an ounce of blood was lost when a double-headed roller and ice were applied, which checked the hæmorrhage.

At 6, p. m., he had an opiate injection, and at 10, p. m., an injection of beef-tea with laudanum. The opium produced a good deal of drowsiness and some stertor. In the course of the next day he had two beef and one thick gruel injection. A draught of citrate of potass was given to allay thirst, and a little chicken-broth was swallowed occasionally. On the 10th, a calomel purge was given. The majority of the sutures were removed on the fourth day. The greater part of the wound united by the first intention, though posteriorly a small aperture remained for some time and gave issue to the saliva. This was finally closed, and he left the hospital apparently well, with the exception of cough, which still continued troublesome. There was remarkably little deformity.

The disease returned in this patient, but we do not at present know the particulars.

*Case* 2. Anne Taylor, æt. 45, admitted March 8th, 1830, under the care of Mr. Brodie.

Great enlargement of the right side of the lower jaw-bone, extending from the second incisor tooth to the condyle, and forming a large globular tumour, occupying nearly the whole of the right side of the face. Tumour extending downwards over upper part of neck—inwards displacing the tongue, and interfering materially with speech and mastication—but chiefly upwards and outwards towards the malar bone. In some parts it is of bony hardness—in others it communicates the sensation of the finger pressing on an elastic gum bottle, and appears to contain fluid. Integuments not at all discoloured—no enlargement of the neighbouring glands—little pain—great numbness about the chin and angle of the mouth. General health good.

First noticed the disease 18 years ago, when it was a small, hard, incompressible lump, immediately over the angle of the jaw. It gave no pain, and its increase was for a long time extremely slow. Six months since, it was not larger than a hen's egg. About that time it began to enlarge very rapidly, and has continued to do so ever since.

March 25th. Operation commenced by an incision, dividing the lip opposite the symphysis, and extending to the upper part of the thyroid cartilage—a second, made from the back of the zygoma to a short distance below the corner of the os hyoides—centres of the two incisions connected by one running transversely across the most prominent part of the tumour. Parts covering the tumour dissected upwards and downwards, so as to form two flaps. In doing this, an opening was made into the tumour, and about two ounces of transparent gelatinous fluid escaped. Several arteries of size requiring ligature—parotid gland pushed upwards, without being much injured. Bone divided at the symphysis with a metacarpal saw. It was now found that the ramus, as high as the condyle, as well as the coronoid process, participated in the disease, the latter projecting considerably beneath the zygoma. The ligaments were divided, and the bone dislocated. On passing the scalpel behind the bone, so as to separate the parts attached to its internal surface, a large artery, supposed to be the internal maxillary, increased in size, was divided; several other vessels were also cut in this situation. The bleeding was suspended by pressure, while the removal of the diseased part was completed. Towards the latter end of the operation the patient became much exhausted; she was allowed to remain in the horizontal position for some time, but, the circulation not being restored, a ligature was placed on the external carotid, about half an inch from its origin, as a security against the recurrence of hæmorrhage. The ligature of the vessel required but little dissection, as it lay pulsating on the surface of the wound. The integuments were brought together by sutures, and some light dressing applied.

*Vespere.* On her removal to bed, she had 20 minims of the liq. op. sed. She vomited in about an hour afterwards. In about three hours, she had in a great measure recovered from the collapse produced by the operation, and there was a slight oozing of blood from the mouth. She had a good night, and next day she was allowed beef tea and arrow-root. On that evening the pulse was 120, with some jerk—skin rather dry—bowels not open since the operation. *V.S. ad* ℥viij. *Injectio c̄ Ol. ricini,* ℥j. *cràs mane.*

In the evening of the 28th she felt chilly, and, on the 29th, she seemed rather depressed. Nearly the whole of the wound appeared to have adhered; the integuments had sloughed in two or three spots. The bowels were disposed to be confined. *Vin. rubri, ad* ℥iv. *Ext. col. c.* gr. v. *Hyd. sub.* gr. iij. *stat.*

On the 30th, a blush of redness, with defined edge, had appeared upon the temple. Pulse 100—skin cool. The erysipelas spread slowly. On April 1st, a slough separated in the site of the angle of the jaw, and left an opening communicating with the mouth. On the 2d, the erysipelas was spreading down the neck—pulse 108 and weak—bowels disposed to be relaxed. Opiates, with aromatics, checked the diarrhœa, but the erysipelas continued to spread; she grew weaker, and had nocturnal delirium. She voided several lumbrici, from the bowels. On the 5th, she was ordered quina, with opium. In consequence of the external opening communicating with the mouth, it became necessary to administer nourishment through the stomach pump. She sank progressively, and at 2, a.m. of the 9th, she died.

*Sectio Cadaveris.* About three ozs. of turbid serum in the cavity of each pleura. At the posterior part of the middle lobe of the right, and inferior lobe of the left lung, several detached, whitish-looking spots, a good deal resembling purulent deposites in their commencement. The substance of the lung surrounding these slightly inflamed.

No other visceral affection.

II. The next case is one of morbid growth, apparently fungoid, of the antrum. No operation was performed, and the case will shew how slowly some of these malignant tumours proceed.

*Case.* Jemima Young, laundress, æt. 21, admitted Sept. 29th, 1830, under the care of Mr. Brodie.

Prominent enlargement of left cheek, about the size of a small apple, and somewhat pointed—tumour reaching from just below the infra-orbital ridge to the gum. In the situation of the infra-orbital fossa, the tumour feels elastic externally, as if the bony wall of the antrum were absorbed. Gum, from left canine tooth to dens sapientiæ, deprived of teeth, rounded, prominent, and elastic—palate on that side somewhat prominent and elastic. By alternate pressure of one finger on the outside of the gum, and another on the palate—indistinct fluctuation, or rather elasticity, perceived; the same on similar pressure of the prominent part of the cheek and gum. No encroachment on the orbit. Prominent part of the cheek painful on pressure—occasionally a little darting pain in the tumour and side of the head. Pulse rather frequent—complexion rather sallow—says her health is good—catamenia regular.

Four years ago, had pains in the teeth of the left side. Extraction was attempted, but the teeth were broken, and parts of them left behind. Sixteen months ago the stumps were taken out, and, about that time, she first noticed enlargement of the gum. Matter followed the removal of the stumps, and she describes a probe as having been passed up to the eye, evidently in the antrum. The matter was discharged for a fortnight, and then the opening closed. It has since been re-established several times by operation, but has always closed spontaneously afterwards. The gum was punctured lately in Guy's Hospital—blood only issued. She was there recommended by Sir Astley Cooper to submit to an operation, but refused, and left the hospital. Six weeks ago, Mr. Brodie again punctured the gum—again blood issued. The tumour has been increasing much lately.

On the 6th October the gum gave way, and according to her account, some matter was discharged. On the 8th Mr. Brodie once more punctured the gum with a lancet, but procured an evacuation of blood only. The divided structure appeared of a gelatinous consistence. The patient was now put on iodine, ten minims of the tincture of which she was ordered thrice daily.

The gum, where it was cut, evinced some disposition to produce a fungus, but this subsided. On Nov. 5th, the dose of the tincture of iodine was increased to 15 minims thrice daily. On the 29th the iodine was discontinued in consequence of its disagreeing with the health. On December 7th, it was resumed in doses of eight minims thrice daily. On the 31st it was again necessary to discontinue it, and to substitute purgatives and leeches to the temples. On the 12th January the iodine was repeated; on Feb. 3d, the dose augmented to 15 minims; on the 6th to 20 minims; and on the 9th one drachm of the ointment of the hydriodate of potass rubbed twice daily upon the tumor. On the 12th, the iodine having again disagreed, was omitted, and not afterwards tried. The further treatment was unimportant.

*March 5th.* Within the last fortnight the tumor has made more progress than it had done for some time previously. Its extension has been chiefly outwards on the cheek. During the last month it has increased so as to reach from the dens sapientiæ to the left lateral incisor, being bisected in the line of the gum by a deepish ulcerated furrow. More of the left side of the bony palate is absorbed, and the tumor displays in the mouth, a shining and smooth appearance.

*May 1st.* A few days ago the poor girl left the hospital. The tumor has been making rapid progress latterly, projecting more on the cheek, and even protruding between the lips. She has suffered from pains in the head, but otherwise the health has been little deteriorated, and she has scarcely lost flesh.

We saw the patient accidentally some months after this. The tumor had increased very little in size since the period of her quitting the hospital. The termination of the case is unknown to us.

### III. POLYPOID GROWTH FROM THE ÆTHMOIDAL CELLS.

*Case.* Caroline Harris, aged between 50 and 60, was admitted July 28, 1830, under the care of Dr. Hewett.

The left side of the face, from the angle of the mouth to the orbit, was tumefied, the soft parts a little thickened, the integument tinged with a slight flush. She complained of pain in the cheek extending to the side of the nose, inner angle of the orbit, and brow. The Schneiderian membrane of both nostrils presented some superficial ulceration. There was considerable purulent discharge from the nose, falling down the posterior nares into her throat, when she lay upon her back. Health tolerably good.

The complaint had begun in the preceding January, when convalescent from a severe attack of erysipelas of the face.

In August she was placed under the care of Mr. Brodie, who immediately punctured the antrum with one end of a pair of scissors, immediately above the first molar tooth. Only fluid issued. Much tumefaction ensued, and for a few days the pain and discharge seemed both relieved. The improvement, however, was temporary, for on Oct. 3, there was more discharge than ever. The gum above the teeth seemed thickened.

*Sept. 29th. Hyd. Sub.*, gr. ij. *Opii*, gr. ½, *bis die.*

*Oct. 4th. Sumat pil. semel die.*

*6th. Sumat pil. alternis diebus.*

*7th.* Mouth very sore indeed. *Garg. alum. Omr. pil.*

*11th. Inf. casarill.* ℥xij. *T. card. c.* ʒj. *M. t. d. s.*

*19th.* During the salivation the pain and discharge diminished in some degree, but both have nearly regained their former state.

*25th. Pil. hyd. sub. c.* gr. v. *o. n.*

*Dec. 9th. Quin. sul.* gr. ij. *Acid. sul. dil.* ♏. iv. *Ex. aq. t.* v.

*Omr. alia.*

*17th. Omr. haust. quinæ.*

Soon after this she was attacked with inflammation and suppuration in and about a bunyon on the great toe. When this was passing away, she was seized with fever of a low type and symptoms resembling those of latent pleuro-pneumonia. After three or four days she died.

On dissection no trace of inflammation was found in the lungs, nor any recent organic change of consequence elsewhere.* Attached to the parietes

* We do not believe that the veins of the limb were examined.

of the æthmoidal cells on the left side was a polypoid growth, organized, not quite so translucent as the gelatinous polypus, not of much magnitude, nor of any regular form.

### IV. Scrofulous Disease of Lower Jaw.

*Case.* Joseph Spilman, æt. 11, admitted May 5th, 1830, under the care of Mr. Brodie.

Lower jaw on right side presenting an equable enlargement, extending from near the angle to near the symphysis—generally hard, smooth, not tender to the touch — in diameter from above downwards about an inch and a half, from before backwards two inches and a half. Within the mouth at the root of the first molar tooth, and at the point of junction between the cheek and gum, an ulcerated opening with rather raised borders, from which pus wells upon pressing the external tumor, and through which a probe passes down to exposed bone. Skin covering the tumor quite healthy. On left fore-arm, over about the middle third of the ulna, hardness and tumefaction, with several ulcerated openings; no exposed bone felt here. A great number of warts upon the hands. Appearance delicate and scrofulous, with a languid circulation.

The tumor on the jaw was of two years' duration. He had observed that pus appeared in his mouth since the last Summer. The affection of the arm commenced nearly at the same time as that of the jaw.

*Tinct. ferri muriatis,* ♏xv. *t. d. Cat. lini. cubito.*

*July* 3*d.* To-day Mr. Brodie enlarged the opening in the mucous membrane, and extracted three pieces of dead bone. On the 23d several other pieces of bone were extracted. The ulcerated opening diminished in size, no more carious bone could be felt, and in September the boy was dismissed the hospital very much improved both locally and generally, but not cured. There was still a considerable degree of thickening about the angle of the jaw, and probably some bone yet to be separated.

We have since heard, but we cannot vouch for the truth of the report, that the boy is dead. We have not been able to learn any particulars.

---

### X. Guy's Hospital.

### Ulcerations of the Face treated by Acids.*

We notice these cases, not because they are possessed of much intrinsic interest, but because one or two remarks are made which ought not to pass unnoticed. The cases are too short to admit of further abbreviation.

*Case.* "Sarah Cobbins, æt. 36, of a sallow, unhealthy complexion, was admitted into Guy's Hospital, under Mr. Key, on the 12th of September, with a deep strumous excavated ulcer of the upper lip, of two years' standing, which had extended to and nearly destroyed the septum nasi: had been under the care of a surgeon in the country during that time, but derived no benefit from the remedies which were employed. She was ordered on her admission Acid. Nitric. Dil. gtt. xl. ex Decoct. Sarsa. ter die sumend.; and the following lotion to be applied to the sore:—℞. Ext. Opii, gr. v. Argent. Nitrat. gr. iij. Aquæ ℥j. M.—which treatment she continued to the 13th of October without any beneficial result. Subsequent to her admission, a small abscess formed on the anterior surface of the cartilage of the nose, which, on being opened, ulcerated, and destroyed a considerable portion of its substance.

Oct. 14th.—Ordered to continue the mixture, with the addition of 10 drops of the Acid. Nitric. Dil. to each dose; and to apply the following lotion to the sores:—

℞. Acid. Nitric.
Acid. Muriat. āā. gtt. xij.
Ext. Opii, ℈j.
Aquæ, ℥vj.

Under the latter treatment, the ulcer has rapidly improved up to the present

---

* Medical Gazette, Dec. 15th, 1832.

time, and she is now (Nov. 30th) sufficiently recovered to be enabled to leave the hospital.

This woman had evidently suffered severely from syphilis; the throat had been formerly ulcerated and the velum injured. Her constitution was now in so impaired a condition, that mercury could not be exhibited, even supposing that the disease in the nose had a venereal disposition. Mr. Key, therefore, put her under a course of dilute nitric acid, which he commonly prescribes when mercury is indicated, but cannot be exhibited, on account of the patient's general ill health. The effect of this remedy, together with the local application of the nitro-muriatic acid lotion has been most satisfactorily shewn in the progressive improvement of health, and the uninterrupted healing action of the ulcer."

We have no means of knowing whether the reporter is correct in attributing the preceding sentiments to Mr. Key. We hope he is not. The gentleman who signs himself W. J. E. observes that the woman had *evidently* suffered severely from syphilis, apparently, because the throat had formerly been ulcerated. We can tell this gentleman, that if there was no other evidence than what he has brought forward, he has very much to learn, and the less he displays what he has learnt, the better. We need scarcely observe that ulceration of the throat is constantly occurring from the administration of mercury, and is cured by withdrawing that medicine. The reporter also observes that, Mr. Key commonly prescribes dilute nitric acid when mercury is indicated, but cannot be exhibited on account of the state of the patient's health. If this be the case we are rather surprised at Mr. Key. From what we have seen of the venereal disease, we must say, that nitric acid has always agreed best when mercury has *not* been indicated. There is no charm about nitric acid, it acts in these, as in other cases, like a tonic, and tonics, as a general rule, are required where mercury is not. If mercury is indicated, and the state of the general health forbids its use, still the plan of treatment, attributed by the reporter to Mr. Key, is a bad one; for the improvement of the health will not remove the indication for mercury, but confirm it. The truth is, that if mercury be *indicated,* nothing else will do; if the general health is bad, it must be improved, but as soon as that improvement has been effected, the indication for mercury will only become more apparent. The whole tenor of the reporter's remarks is based on a false notion of the venereal disease.

Case 2. "*Strumous Ulcers commencing in Ecthyma.*"

"Susan Burford, æt. 18, admitted under Mr. Key, October 25th, affected with strumous cachectic ulcers on both of the thighs and legs, of three months' standing. The patient is of an unhealthy exsanguineous appearance, subject to hysteria, and has had her catamenia absent four months. On her admission, the surfaces of the sores were covered by a thick brown incrustation, which being removed, exposed unhealthy indolent ulcers. The first remedies employed were a poultice, to remove the incrustations, and, internally, infus. cascarillæ, with carbonate of soda, bis die, and hydr. c̄ cretâ, gr. ij. omni nocte, in starch. These remedies were continued till the 29th, when the sores became uncovered, and the following lotions prescribed.

Acid. Mur. gtt. xij.; Acid. Nitric. gtt. xij.; Ext. Opii, ℈j.; Aq. Puræ, ℥vj. ft. lotio.

This was applied to the right leg, on which were seated the deepest ulcers.

To the other leg the iodine lotion, consisting of—

Iod. gr. ij.; Potass. Hydriod. ℈j.; Aq. Puræ, ℥vj. ft. lotio.

At the end of a week the gums became affected by the hyd. c̄ cretâ, which gradually increased till November 12th, when it was thought expedient to administer the mercurial every other night only, being sufficient to keep the constitution under its influence. The lotions have been persisted in up to the present period, with the greatest benefit, all the ulcers having nearly healed."

This case is headed, and we suppose was considered " strumous ulcers commencing in ecthyma." Yet the patient was salivated and maintained so. We would ask, for which affection of this bi-corned malady, salivation was deemed expedient—for the struma or the cachexia? We had deemed that salivation was not adapted to, nor by good surgeons adopted in, either. Let the reporter answer.

" These ulcers, at her admission into the hospital, bore the character of rupia, and the scabs had both the form and the dirty brown tint characteristic of syphilitic action; but on closely questioning her, there was no reason for believing that she had had any symptoms to warrant this suspicion. They were, therefore, considered and treated as strumous ulcers, and the mildest form of mercurial alterative, with alkali, and the cascarilla, were administered, according to the plan which Mr. Key usually finds most successful in treating this kind of sore."

So the salivation, by the " mildest form of mercurial alterative," was employed for the strumous taint, and, what is more, Mr. Key finds this the most successful plan of treating this kind of sore. The reporter has contrived to convey a great deal of information in a very words:—first, that there is " a form and a dirty-brown tint in a rupia scab, characteristic of syphilitic action—and secondly, that the hydrarg. c̄ cretâ is the mildest form of mercurial alterative. The first piece of information is quite new to us; who had thought that rupia was a cachectic disease, and not essentially syphilitic. The second is also new, for we had imagined that the Plummer's pill was a milder alterative than the hydrargyrus c̄ cretâ. We live and learn.

---

### XI. London Hospital.

### Osteo-Sarcoma—Removal of the Superior Maxillary and Malar Bones.*

In the present number of this Journal will be found the details of three cases of " osteo-sarcoma," for which operations were performed without success. The present will unfortunately swell the list.

Eliz. B. æt. 48, admitted Sept. 8th, under Mr. Scott. There was a large " osteo-sarcomatous tumour," principally in the situation of the left malar bone, and on removing the last molar tooth from its socket, the probe was readily passed into the antrum. The palatine process of the superior maxillary bone seemed sound, and resisted the passage of a sharp-pointed probe. A grooved needle was introduced beneath the upper lip into the swelling, and on withdrawing it the groove was found filled with a thick medullary substance. The health was indifferent. The disease had commenced with pain six months previously.

On Sept. 12th, the operation was performed by Mr. Scott. It was begun by tying the external carotid. An incision was now made, extending from the angle of the mouth obliquely upwards, and backwards towards the zygoma, and the integuments were dissected upwards from the surface of the tumor. The eye was then separated from its loose cellular connexion with the floor of the orbit, and the left ala of the nose detached. With the strong cutting plyers, the malar and temporal bones were disunited at the zygoma. The malar was next detached from the frontal bone with the same instrument, the nasal process of the superior maxillary bone cut through, and lastly, the two maxillary bones separated at the longitudinal palate suture. The tumor was then readily dissected away—the chasm filled with lint—and the edges brought together by sutures. The wound united, with the exception of the upper part, the ligature came away on the 12th day, and the health seemed at first improved. Subsequently, cough and hectic came on, and the patient died on the 20th October.

On examination there was necrosis of a portion of the zygoma, but no return of the disease in the face. The lungs were studded with " tubercles." The disease was medullary; it had des-

* Med. Gazette, Jan. 12th, 1833.

troyed the whole of the malar bone, and seemed to have grown from the outer wall of the antrum.

---

### XII. MIDDLESEX HOSPITAL.

#### I. SIR CHARLES BELL ON CONTRACTION OF THE PREPUCE.*

This excellent physiologist and able surgeon was called to Seven Oaks to see a young boy, who had extreme difficulty of making water. This was found to be owing to contraction of the orifice of the prepuce, and on slitting this up the relief was complete, and the boy exclaimed, "I shall now be able to piss against the wall like papa."

A boy, æt. 5, was admitted into the Middlesex Hospital, with the scrotum enormously distended and sloughing at its posterior and inferior part—the integuments of the penis distended—and phymosis. The mother declared that the parts had only begun to swell two days previously, and this was all the history that could be procured. Scarifications were made in the scrotum and urinous fluid evacuated. On the evening of the second day after his admission he died.

On examination the urethra was found quite free from contraction. The orifice of the prepuce was closed, and ulceration was found extending back into the cellular substance of the integuments of the penis;—ulceration existed at the angle made by the prepuce and frænum. There was no other disease.

Sir C. Bell observes that the second case displays an ulterior degree of the same affection that existed in the first. The prepuce, distended in the act of micturition, becomes successively irritated inflamed, ulcerated; the urine escapes into the cellular membrane; and the consequence is sloughing of the latter. Sir C. Bell divides natural phymosis into four varieties, or degrees.

"You have before you, then, gentlemen, the effects of the first or greatest degree of stricture in the prepuce, or natural phymosis. You perceive that the natural phymosis is followed by inflammation and thickening, that the orifice becomes smaller through the thickening of the margins of the hole, and you see the unhappy consequences. Marking this as the first or the greatest degree of stricture of the prepuce, we come, in the *second* place, to common phymosis, where the prepuce permits the urine to flow freely, and consequently without distending it. Now the prepuce not being washed by the urine, a foul secretion from the glands about the corona-glandis collects within it, and so inflammation and thickening, with the discharge of purulent matter, and sometimes ulceration, follow. The third instance to be noticed is where there is only that degree of narrowing of the prepuce that it prevents the foreskin from being drawn freely over the glans; and when by accident the prepuce is so drawn, it is in danger of producing paraphymosis. There is a *fourth* kind, and it is to it that I will particularly draw your attention, because the effects of it are not noticed, and yet they are very terrible, so that patients are almost inclined to part with life from the distress that it produces. This distress does not arise from the urinary organs: the difficulty is not in discharging the urine; there is just that degree of tension at the margin of the prepuce that it can be drawn over the glans, and then it produces a a stricture; but it is only during priapism, and emission is obstructed, not urine; and the consequences are excessive irritation and great distress of body and mind. Thus you are acquainted with the first degree of stricture; you see that the second is where there is an accumulation of the secretion and consequent inflammation caused by the stricture; the third degree is where there is danger of paraphymosis; and a fourth degree is where the skin can be drawn backwards and forwards, but is too narrow for the distension of the penis in a state of priapism, and then it produces stricture, not upon the urethra as the canal for the urine, but as it belongs to the organs of generation."

---

* Med. Gazette, Jan. 12th, 1833.

Sir Charles Bell says nothing about the treatment of these cases. We will supply the deficiency. Slit up the prepuce at the distance of about a quarter of an inch from the frænum, and all inconvenience vanishes. We have done this in several instances of the last affection at which Sir C. glances, and the relief was complete and permanent. The lecturer goes on to notice another case.

*Case.* A man, æt. 42, was admitted with the prepuce and integuments of the penis enormously enlarged, and firmly so—a small abscess, opening externally, anterior to the scrotum—issue of the urine through the inner membrane of the prepuce, having arrived there by travelling between the skin and body of the penis—and, finally, a narrow stricture. The patient had had acute gonorrhœa one year previously; the difficulty of making water for six months.

Sir C. Bell remarks that, during the gonorrhœa, one of the lacunæ had become inflamed, formed an abscess, urine got into it, ulceration occurred, and the urine passed between the skin and body of the penis to the inner prepuce, at the angle, through which it escaped by another process of ulceration. The stricture was occasioned by the thickenings, &c. round the lacunar abscess.

"This inflammation of the lacunæ is a very troublesome complaint, and will sometimes continue long after the gonorrhœa has entirely subsided, being attended with tumor and thickening of the integuments opposite to the part. It is to be treated, first, by all the possible means of subduing inflammation; then by an injection; I have touched the point with caustic from the inside, and have sometimes been forced to make an incision on the outside. We shall be satisfied in the present case with the use of the bougie and the gradual dilatation of the thickened membrane of the urethra. Respecting the use of injections, let me remind you, that an injection for gonorrhœa, comparatively mild and innocent, will be made most effectual, if you take care that the patient compress the urethra just anterior to the scrotum, and also that he prevent the injection from coming back on the syringe. This method of injecting the canal will produce fulness and tension in the part which is the seat of the original specific inflammation of gonorrhœa. You may thus manage a gonorrhœa by an injection which is comparatively mild, and without the necessity of increasing its strength so as to endanger the bringing on of inflammation from that new cause: and this manner of distending the urethra is particularly necessary when the inflammation fixes upon the lacunæ, for without it the astringent does not reach the diseased surface."

We may mention, by the way, that if the lacunar abscess is opened from without, it is apt to occasion a very troublesome urinary fistula.

### II. Femoral Hernia, with Double Hernial Sac.*

*Case.* M. M. æt. 37, admitted April 18th, vesp., with femoral hernia on the right side. Great pain and tension of the abdomen—stercoraceous vomiting—pulse small, quick, sharp—countenance pallid, anxious. She had been ruptured for 17 years, and the tumour had never entirely disappeared—the hernia had been down for five days—the bowels not relieved for six. She had been treated for peritonitis with leeches and a blister.

The tumour being irreducible by the taxis and warm bath, Mr. Tuson, after consultation with Mr. Arnott, determined to operate, which he did at 1, a.m. of the 19th. The sac being opened, half an ounce of serum escaped, and a curious tumour was observed. Appearing to be thick and vascular, it was thought to be part of the large intestine. The stricture was divided, by cutting the ligament of Gimbernat, and the tumour was compressed, but a considerable portion remained irreducible. An incision was made into it, when it was found to be the true sac, with the intestine within it, but not the slightest

* Lancet, May 25th, 1833.

appearance of fluid. The intestine was highly vascular—it was returned. *Castor oil immediately—injection in three hours.*

10, a.m. Bowels not opened—much swelling over abdomen—pulse 100. *Enema. H. sal.* c̄ *May. sul.* ℥j. 4*tis horis.* At noon, great pain and tension of abdomen—anxious countenance—pulse 120. *Hirud.* xvj. *Cal.* gr. ij. 8*vis hor.* The bowels had been moved twice, and were opened again. At 10, p.m. she was relieved, but complained of sinking and want of sleep. *Haust. morph. acet.* At 1, p.m. of the 20th, more tenderness of abdomen and anxiety—pulse 100, full. *Hirud.* xij. 8, p.m. *Castor oil.* From this time, the symptoms continued unmitigated. The treatment consisted in calomel and Dover's powder—saline purgatives—bleeding to 12 ozs. on the 21st—bleeding to 4 ozs. on the 22d, with 12 leeches. At 11, a.m. of this day she died.

*Inspection.* Stomach and intestines much distended with fluid and flatus, and generally inflamed. The intestine which had been in the sac had never recovered itself; at one part it had sloughed, and a portion of its contents were in the abdomen; the part opposed to the stricture had given way.

Perhaps the treatment might have been more depletory in the first instance, with advantage.

